# Chemical sensing in two dimensional porous covalent organic nanosheets†
†Electronic supplementary information (ESI) available: See DOI: 10.1039/c5sc00512d



**DOI:** 10.1039/c5sc00512d

**Published:** 2015-04-29

**Authors:** Gobinda Das, Bishnu P. Biswal, Sharath Kandambeth, V. Venkatesh, Gagandeep Kaur, Matthew Addicoat, Thomas Heine, Sandeep Verma, Rahul Banerjee

**Affiliations:** a Physical and Materials Chemistry Division , CSIR-National Chemical Laboratory , Dr HomiBhabha Road , Pune-411008 , India . Email: r.banerjee@ncl.res.in ; Fax: +91-20-25902636 ; Tel: +91-20-25902535; b DST-Thematic Unit of Excellence on Soft Nanofabrication , Indian Institute of Technology Kanpur , Kanpur-208016 , UP , India; c Centre for Functional Nanomaterials , Engineering and Science , Jacobs University Bremen , Research III, Room 61, Campus Ring 1 , 28759 Bremen , Germany; d Academy of Scientific and Innovative Research (AcSIR) , New Delhi , India

## Abstract

Two new imide-based crystalline, porous, and chemically stable covalent organic frameworks (COFs) (**TpBDH** and **TfpBDH**) have been successfully synthesized employing solvothermal crystallization route. Furthermore, thin layered covalent organic nanosheets (CONs) were derived from these bulk COFs by the simple liquid phase exfoliation method. These 2D CONs showcase increased luminescence intensity compared to their bulk counterparts (COFs). Notably, **TfpBDH**-CONs showcase good selectivity and prominent, direct visual detection towards different nitroaromatic analytes over **TpBDH**-CONs. Quite interestingly, **TfpBDH**-CONs exhibit a superior “turn-on” detection capability for 2,4,6-trinitrophenol (TNP) in the solid state, but conversely, they also show a “turn-off” detection in the dispersion state. These findings describe a new approach towards developing an efficient, promising fluorescence chemosensor material for both visual and spectroscopic detection of nitroaromatic compounds with very low [10^–5^ (M)] analyte concentrations.

## Abstract 2

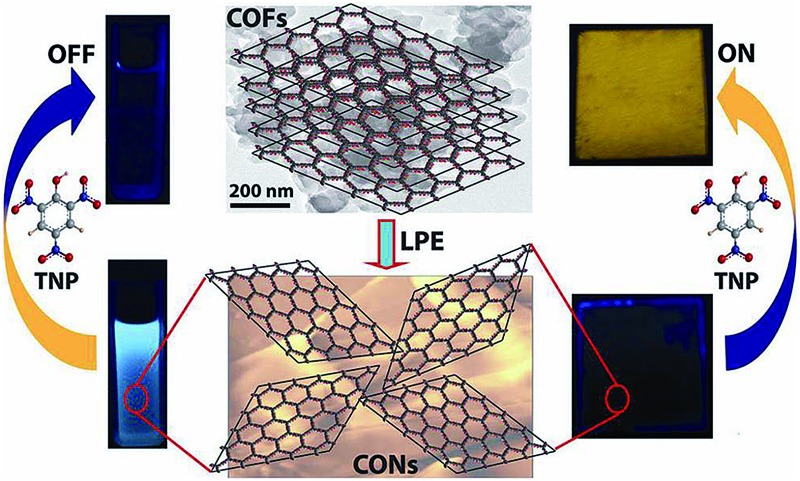
Covalent organic nanosheets (CONs) were synthesised from imide functionalised COFs. **TfpBDH**-CONs exhibit a "turn-on" detection capability for 2,4,6-trinitrophenol in the solid state, but show a "turn-off" detection in the dispersion state.

## Introduction

Chemical sensing using porous materials, especially Metal–Organic Frameworks (MOFs)^1^ and Porous Organic Frameworks (POFs)^2^ has picked up considerable attention recently. An important feature of these frameworks is their flexible and adjustable pores in which guest molecules can freely interact with the pore walls.^2e–g^ However, the sensitivity and selectivity of detection in these materials is generally not sufficient, either due to the poor analyte–host interactions inside the amorphous porous organic frameworks, or due to the chemical instability of the crystalline metal–organic frameworks. Hence, a chemically stable, crystalline and luminescent porous material with electronically adjustable π-conjugation is an attractive choice for acting as an efficient chemical sensor.

Covalent Organic Frameworks (COFs)^3^ are one such class of porous materials that, like MOFs, exhibit a well-defined and predictable crystalline network. A large number of COFs have been synthesized over the past few years, but these materials have only rarely been explored as chemosensors^3*h*^ owing to their chemical instability in aqueous and acidic/basic medium. With this in mind, we have synthesized two imide based COFs, each possessing a two dimensional layered structure, that exhibit porosity, crystallinity and chemical stability. Since imide functionalized linkers show good photophysical properties, one might predict that these COFs would act as efficient chemical sensors.^4^ However, the bulk COFs exhibit only moderate chemical sensing ability due to the aggregated π-stacked layers, poor electron mobility and ineffective interaction with analytes. In order to address these shortcomings, we exfoliated the aggregated π-stacked COF layers using the Liquid Phase Exfoliation (LPE) method^5a–c^ to produce Covalent Organic Nanosheets (CONs). Since π–π interactions between the stacked layers are considerably weakened in these two dimensional covalent organic nanosheets (2D CONs), we anticipated that these CONs might show superior chemical sensing capabilities compared to the bulk COF. There have been a few reports on the growth of thin COF layers on 2D surafaces,^5*i*^ but very little effort has been made to isolate such 2D materials in bulk quantities and to investigate their usefulness. In this paper, we show that these exfoliated CONs exhibit highly selective and visual detection of 2,4,6-trinitrophenol (TNP), over other nitroaromatic analytes such as 2,4,6-trinitrotoluene (TNT), 2,6-dinitrophenol (DNP), 2,6-dinitrotoluene (DNT), and 2-nitrophenol (NP), *via* a “turn-on” mechanism in the solid state and a “turn-off” fluorescence quenching in the dispersed state. Furthermore, the sensitivity of the CONs towards nitroaromatic analytes detection is ∼10 fold (turn-on) and 63% (turn-off) higher with respect to the bulk COF, even at a very low [10^–5^ (M)] analytes concentration.

## Experimental section

### General procedure for the synthesis of **TpBDH**, **TpBDH**-CONs, **TfpBDH** and **TfpBDH**-CONs

The COFs reported in this paper [**TpBDH** and **TfpBDH**] were synthesized using a standard solvothermal protocol reported previously.^6^ The detailed synthesis of materials and experimental methods can be found in Section S1 and S2, ESI.† In brief, 1,3,5-triformylphloroglucinol (**Tp**) (0.3 mmol, for **TpBDH**) or 1,3,5-tris(4-formylphenyl)benzene (**Tfp**) (for **TfpBDH**) and pyromellitic-*N*,*N*′-bisaminoimide (**BDH**) (0.45 mmol) can be allowed to react solvothermally in the presence of 1,4-dioxane, *N*,*N*′-dimethylacetamide (DMA) and 6 M acetic acid (1 : 2 : 0.3) in a pyrex tube at 120 °C for 3 days. After Soxhlet purification, these COFs were dried at 180 °C under vacuum for 12 hours to give a deep red/gray colored powder in ∼80% isolated yield. To produce **TpBDH** and **TfpBDH** CONs, 50 mg of as-synthesized COFs was placed in 50 mL of isopropyl alcohol (IPA) and sonicated at room temperature for 45–60 minutes. The resulting suspension was centrifuged at 1000 rpm for 5 minutes. After complete evaporation of solvent, the residue of the material transferred from the settled solids to the solution as a result of sonication was obtained as CONs in ∼4 wt% isolated yield.

### Description of turn off and turn on sensing experiments

Different concentrations, [0 to 5.4 × 10^–5^ (M)] of analyte (TNP, TNT, DNP, DNT and NP) were added to a homogeneous dispersion of CONs (1 mg in 3 mL of IPA). The fluorescence intensity of the CONs decreased with the increasing concentration of TNP; however, a slight decrease was observed for the other analytes. Secondly, the turn on sensing ability of these isolated CONs was ascertained by filter paper strip assay experiments.^7^ Test strips were prepared by drop casting the homogeneous suspension of CONs in IPA (1 mg in 5 mL) on a strip of filter paper (2 cm × 2 cm), then dried under vacuum at room temperature to obtain the fluorescent paper sensor. Upon addition of 200 μL [1 mg in 100 mL, 5.4 × 10^–5^ (M)] of TNP in IPA onto the test strip, the color of the strip immediately changed from blue to yellow (under 365 nm UV lamp), which can also be visually observed with the naked eye. The same experiment was performed with the other nitroaromatic compounds; TNT, DNP, DNT and NP, but unlike TNP, they did not show any pronounced sensing response.

## Results and discussion

**Fig. 1:**
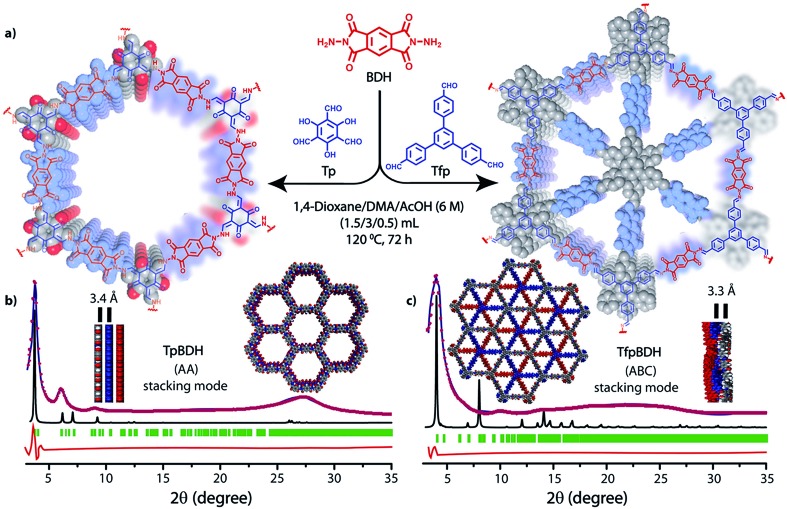
(a) Schematic representation for the synthesis of **TpBDH** and **TfpBDH** [one unit of a space filling model of **TpBDH** and **TfpBDH** is shown in the inset of the chemical drawing model]; (b and c) The experimental PXRD patterns (blue) compared with simulated (eclipsed; black) and Pawley refined difference between experimental and calculated data (red) [*R*_p_ = 1.94%, *R*_wp_ = 4.6% and *R*_wp_ (w/o bck) = 4.33% for **TpBDH** and *R*_p_ = 2.57%, *R*_wp_ = 3.08% and *R*_wp_ (w/o bck) = 3.85% for **TfpBDH**]; for **TpBDH** and **TfpBDH** respectively [inset models showing the eclipsed AA and staggered ABC stacking of consecutive 2D layers of **TpBDH** and **TfpBDH** ^6*c*^ respectively].

**Fig. 2:**
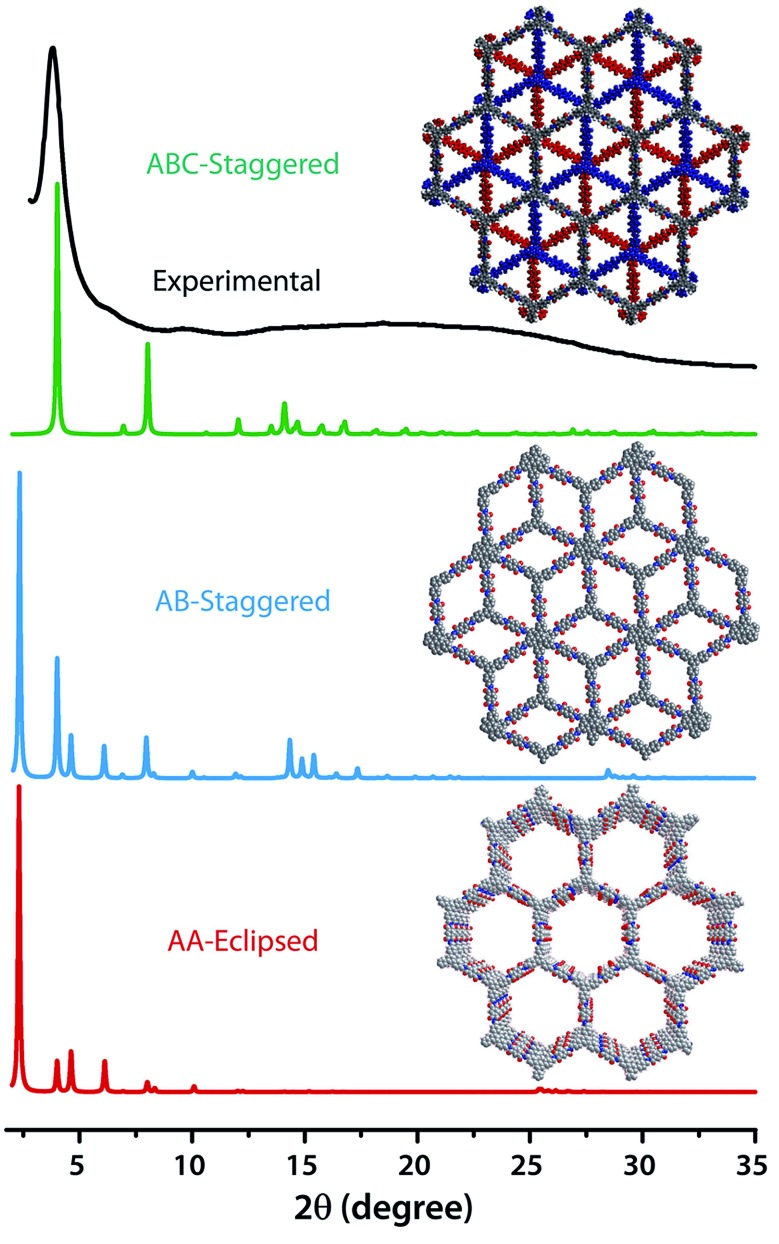
The comparison of PXRD patterns between possible AA, AB and ABC staking models of **TfpBDH**.^6*c*^

The PXRD patterns of **TpBDH** and **TfpBDH** showed intense peaks at 2*θ* ∼3.3°and ∼3.8°, which correspond to the reflection from (100) planes (Fig. 1b and c). In addition to that, **TpBDH** exhibited two minor peaks at 2*θ* ∼5.8° and ∼8.7°; which could be ascribed to (110) and (200) plane reflections respectively. A broad diffuse peak appears at 2*θ* ∼27.3° for **TpBDH** and between 2*θ* ∼15.3° to 27° for **TfpBDH**. This broad peak is mainly due to the reflection from the (001) plane. In order to elucidate the probable structures of these imide based COFs, all possible 2D eclipsed (AA) and staggered (AB and ABC) models were built using AuToGraFS and optimized by the SCC-DFTB method.^8^ The best fitting of the simulated and experimental PXRD determined the probable structure of these imide based COFs. The two COFs adopt a *P*1 space group with unit cell parameters, *a* = 44.00 Å, *b* = 44.02 Å, *c* = 6.6472 Å (for **TfpBDH**) and *a* = 29.9 Å, *b* = 27.9 Å, *c* = 3.3 Å (for **TpBDH**) (Section S3, ESI†). The π–π stacking distance between two successive COF layers was found to be ∼3.4 Å and ∼3.3 Å respectively for **TpBDH** and **TfpBDH**. The experimental PXRD pattern of the **TpBDH** is in good agreement with the simulated pattern from the eclipsed (AA) stacking model. However, **TfpBDH** showed an unusually poor PXRD pattern unlike other hexagonal COFs reported so far.^6^ All possible models related to stacking arrangements were built for **TfpBDH** COF to establish the stable structure *via* rigorous structural modelling and Pawley refinement on the AA, AB and ABC orientations, correlating the experimental and calculated PXRD profiles (Fig. 1 and 2). As a result, we found that the experimental PXRD profile of **TfpBDH** roughly fits with the simulated ABC stacking mode only and not with the AA and AB or any slipped AA or AB structures. Similarly the refinement values [*R*_p_ = 2.57%, *R*_wp_ = 3.08% and *R*_wp_ (w/o bck) = 3.85%] are found to be minimum for ABC compared to AA or AB model. The results of all refinement and the statistics for the various stacking arrangements are elaboratated given in Section S3, ESI.† On the other hand the pore size distribution peak maxima obtained for **TfpBDH** (1.6 nm) from the N_2_ adsorption experiment is close to the theoretical (ABC) pore diameter (∼1.2 nm) and that further support the predicted ABC stacking model rather than AA model (∼3.4 nm) and AB (∼2.0 nm). Although, the first experimental PXRD peak (2*θ* ∼ 3.8°) of **TfpBDH** matches well with the simulated ABC stacking mode, but the peaks at 2*θ* ∼8°, ∼15° and at higher angles (27.5–32.5°) are unidentified and appear as a broad hump. From the theoretical calculations it was found that AA is the most thermally stable form based on stacking energy (Table S4, ESI†). However, the pore size given by the N_2_ adsorption experiment better supports assignment of the ABC structural model. Notably, owing to the very poor crystallinity of **TfpBDH**, our prediction of ABC model for **TfpBDH** is speculative and therefore it can also be termed as a two dimensional porous polymer. Needless to mention we have tried different solvent combinations to synthesize **TfpBDH** but even after several attempts we were unable to improve the crystallinity (Fig. S3, ESI†).

The FT-IR spectra of **TpBDH** and **TfpBDH** indicate the absence of the –NH stretching band of **BDH** at ∼3339 cm^–1^, the carbonyl (–C=O) stretching band of **Tp/Tfp** at 1639 cm^–1^ and at 1689 cm^–1^ respectively. This indicates the complete consumption of the starting materials, as well as the formation of new bonds (Section S5, ESI†). A strong peak for –C=C at ∼1583 cm^–1^ for **TpBDH** appears as a result of enol to keto tautomerism. This peak is merged with the –C=O stretching frequency (∼1609 cm^–1^) of the keto group present in the framework. The appearance of two peaks at ∼1445 and ∼1256 cm^–1^ correspond to the aromatic –C=C and –C–N bond in the keto form of **TpBDH**. However, the band at ∼1604 cm^–1^ for the FT-IR spectra of **TfpBDH** is due to the presence of the –C=N bond, which further confirms the covalent bond formation between the aldehyde group of **Tfp** and the amino group of **BDH**. As evidenced from FT-IR spectroscopy (Fig. S4 and S4a, ESI†), **TpBDH** exists in the keto-enamine form resembling our previously reported COFs^6^ where as **TfpBDH** exists purely in the imine form.

Further support for the local structure of these imide based COFs is given by ^13^C CP-MAS solid state NMR spectroscopy. **TpBDH** showed a clear NMR signal at 182.6 ppm corresponding to the carbonyl (–C=O) carbons of the keto form (Fig. S5, ESI†). However, the resonance of carbonyl carbons of the **BDH** linker unit appears at 163.8 ppm. The peak at 152 ppm confirms the presence of the –C–N bond, instead of the –C=N bond (∼165 ppm), which would have been a signature peak if **TpBDH** had been existing in the enol form. For **TfpBDH**, the presence of the –C=N bond was established by a characteristic resonance signal at 158.7 ppm. As depicted in Fig. S6, ESI;† **TfpBDH** showed a down field resonance at 162.7 ppm for the carbonyl carbon (–C=O) which confirms the presence of the imide (**BDH**) linker unit. The assignment of the peak patterns of both **TpBDH** and **TfpBDH** is consistent with their corresponding monomer compounds made for comparison (Fig. S5 and S6, ESI†).

**Fig. 3:**
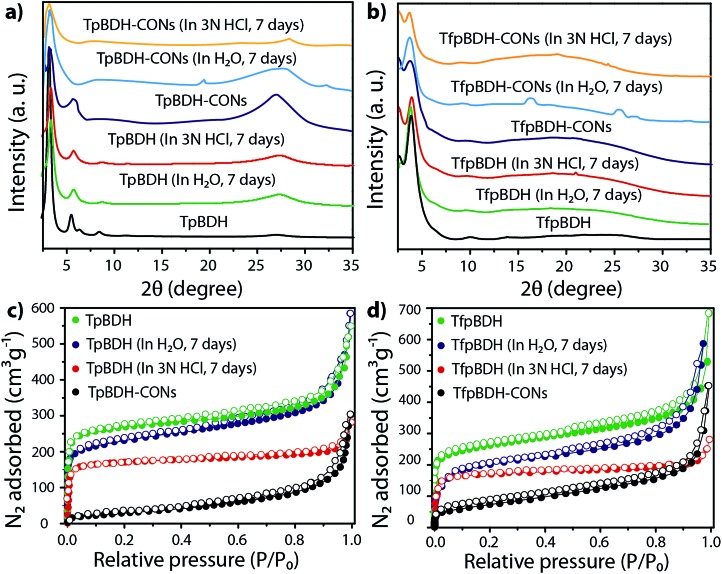
(a and b) PXRD patterns before and after chemical stability tests of **TpBDH**, **TpBDH**-CONs; **TfpBDH**, and **TfpBDH**-CONs respectively; (c and d) N_2_ sorption isotherms; shows the chemical stability of **TpBDH**, **TfpBDH** in water and 3 N HCl for 7 days.

The external morphology of these imide based COFs has been verified using SEM and TEM techniques. The SEM images of **TpBDH** indicated that several COF layers agglomerate to construct a flower-like morphology (Fig. S10(a), ESI†), while for **TfpBDH**, agglomeration of irregular shaped layers with rough surface was observed, (Fig. S10(b), ESI†). We have performed the TGA analysis under the flow of N_2_ gas to check the weight loss (%) of these COFs at high temperature. As observed, the **TpBDH** COF retain the plateau up to ∼394 °C, whereas **TfpBDH** showed similar behavior up to ∼386 °C (Fig. S9, ESI†). N_2_ adsorption isotherms were collected to determine the architectural rigidity and permanent porosity of these COFs at 77 K. Both **TpBDH** and **TfpBDH** showed type II isotherms with reversible adsorption and desorption profiles. The Brunauer–Emmett–Teller (BET) surface areas were found to be 642 and 738 m^2^ g^–1^ (Fig. 3c and d) with pore-size distributions peak maxima at 2.4 and 1.6 nm for **TpBDH** and **TfpBDH** respectively calculated on the basis of nonlocal density functional theory (NLDFT) method (Fig. S7 and S8, ESI†). Although the pore size distribution peak maxima obtained for **TfpBDH** (∼1.6 nm) is slightly higher than the theoretical pore size value (1.2 nm), that further support the predicted ABC staking model rather than AA model (∼3.4 nm). The relative PXRD peak intensity and peak positions of **TpBDH** and **TfpBDH** remained identical after 7 days of water and acid (3 N HCl) treatment, indicating the high chemical stability of these COFs (Fig. 3a–d). The existence of enol-to-keto tautomerization provides the chemical stability for **TpBDH**, whereas the chemical stability in **TfpBDH** is possibly due to the presence of imide functionalities near the imine bonds, which decreases the electrophilicity of –C=N bonds through the mesomeric effect.^11^ The surface area of acid treated **TpBDH** decreases slightly (642 to 517 m^2^ g^–1^), whereas the water treated sample holds the usual surface area (642 to 627 m^2^ g^–1^). However, for **TfpBDH**, the values decrease significantly for both water and acid treated samples [from 738 to 541 m^2^ g^–1^ (for water) to 375 m^2^ g^–1^ (for acid)]. This could be due to the partial degradation, pore blockage or delamination when exposed to acid (3 N HCl) and water for a longer period (7 days).

**Fig. 4:**
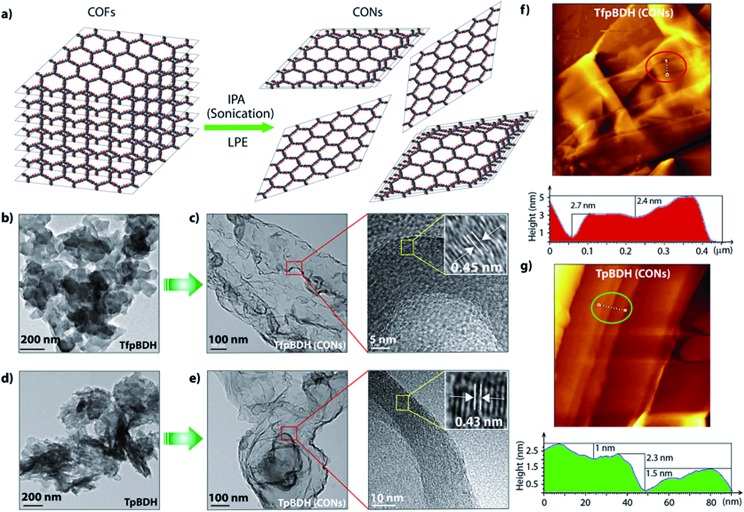
(a) Schematic representation of COFs to CONs formation; HR-TEM images of (b and d) **TfpBDH** and **TpBDH**; and (c and e) **TfpBDH**-CONs and **TpBDH**-CONs; (f and g) AFM images of **TfpBDH**-CONs and **TpBDH**-CONs with corresponding height profiles (the height profiles, colour-filled red and green, are measured along the corresponding tracks inside the circle shown in the AFM images).

Since **TpBDH** and **TfpBDH** contain electron rich triphenyl/keto (donor) and electron deficient imide (acceptor) moieties, we decided to check their nitroaromatic analyte sensing ability. However, the sensitivity and selectivity was found to be quite low for bulk COFs, due to the extensive aggregation of layers, which reduces the available electrons for analyte-COF interaction. Hence, we decided to exfoliate these COFs into 2D CONs to minimize the aggregation and maximize the availability of electron density among the layers.^5^ One-step liquid phase exfoliation (LPE) was undertaken in isopropyl alcohol (IPA) at room temperature to synthesize thin 2D CONs (Fig. 4a). HR-TEM and AFM analyses confirm their typical flat thin 2D-nanosheet structures (Fig. 4b–g). Both **TpBDH** and **TfpBDH** CONs exhibit wrinkles and folding with well-resolved 0.45 nm periodic lattice fringes (Fig. S13 and S14, ESI†). AFM analysis indicates that the heights of **TpBDH**-CONs and **TfpBDH**-CONs are in the range of ∼1.5–5.1 nm, indicating that these CONs are composed of ∼5–15 stacked COF layers (Fig. 4f, g and S15, ESI†). Furthermore, the PXRD patterns of these CONs are identical to that of the parent COFs with a decrease in intensity of the first peak (100) and a broadening of peak at the wide angles (001) (Fig. 3a and b). Such peak broadening and reduced intensity phenomenon has previously been observed for thin layered COF materials and CONs.^5d,6b^ Further, due to exfoliation, the long-range pore structure of the COFs is disturbed and only shallow pores remain accessible for N_2_ sorption resulting in only moderate surface area values for these CONs [270 m^2^ g^–1^ for **TfpBDH** and 112 m^2^ g^–1^ for **TpBDH**] (Fig. 3c and d). FT-IR and TGA also confirms the retention of structural integrity and thermal behaviour (**TpBDH**-CONs and **TfpBDH**-CONs are stable above 380 °C) of these CONs similar to their bulk counterparts (Fig. S9, ESI†). As anticipated, these CONs were found to be highly stable in water, acid (3 N HCl) and even in various organic solvents for 7 days (Fig. 3, S17 and S18 ESI†).

We examined the photoluminescence (PL) spectra of these COFs and CONs reported in this paper with different nitro aromatic analytes *i.e.* TNP, TNT, DNP, DNT and NP. Surprisingly, **TpBDH** and **TpBDH**-CONs do not showed any PL activity, as in both **TpBDH** and **TpBDH**-CONs there exist enol–keto tautomerization^6^ which leads to the disturbance in π-conjugation and “switches off” the fluorescence.

**Fig. 5:**
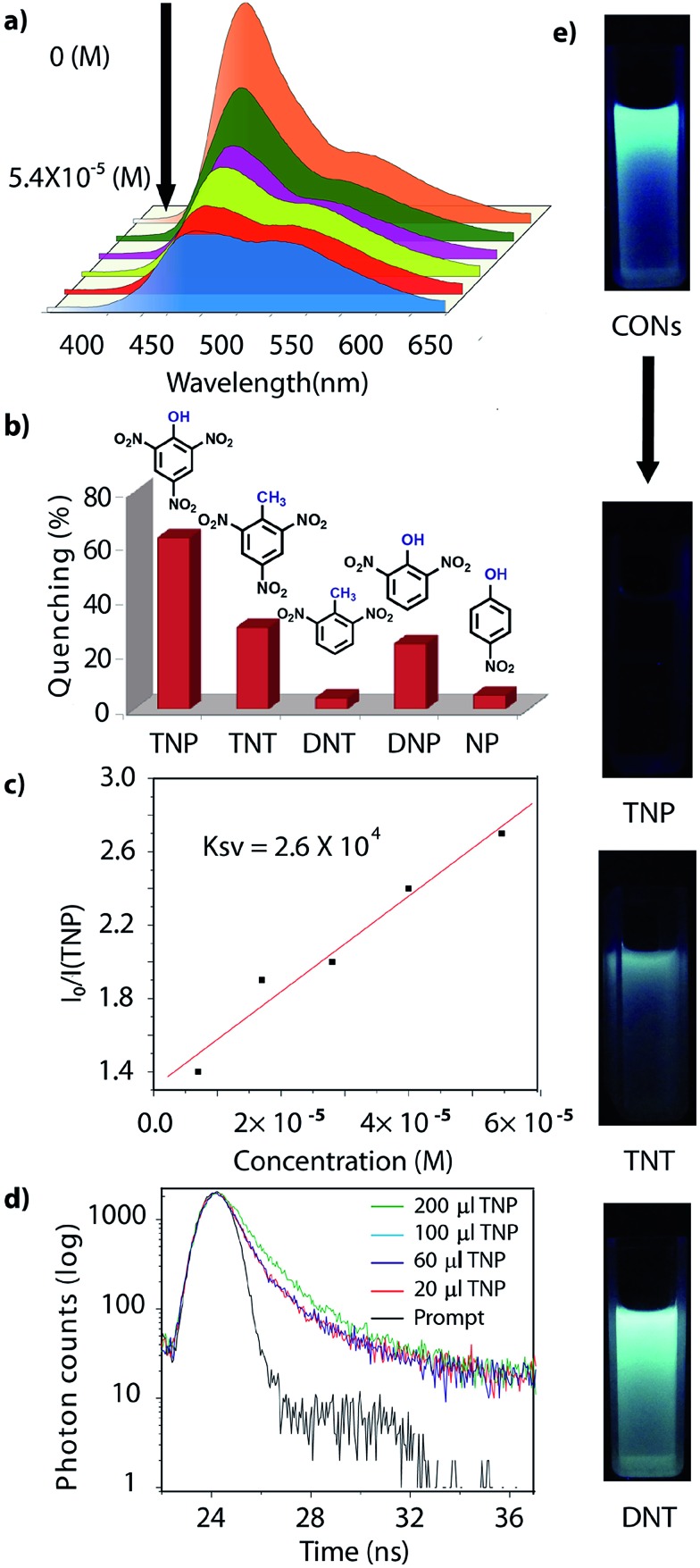
(a) Turn off sensing (PL quenching); (b) degree of fluorescence quenching of **TfpBDH**-CONs with analytes; (c) Stern–Volmer plots for the quenching of CONs by TNP; (d) fluorescence decay profile (*λ*_ex_ = 365 nm and *λ*_em_ = 447 nm) of **TfpBDH**-CONs, in the presence of different analytes; (e) digital photographs of the turn off sensing of analytes under UV light (*λ*_ex_ = 365 nm).

However, due to the presence of extended π-conjugation and donor–acceptor charge transfer, **TfpBDH** and **TfpBDH**-CONs, showcase enhanced luminescence compared to **TpBDH** and **TpBDH**-CONs. Further, we found that **TfpBDH**-CONs exhibit an intense PL peak (∼6–10 times intensity) in both solution as well as solid state compared to the bulk COFs (Section S13, ESI†). **TfpBDH**-CONs showed a slight blue shift (∼20 nm) in the PL spectra with reference to the bulk COF samples.^9a,b^ The HOMO–LUMO gap of the exfoliated **TfpBDH**-CONs (Δ*E* = 1.71 eV, calculated from the UV-visible spectra) is found to be larger than that of bulk COF samples (Δ*E* = 1.14 eV), which clearly justifies the blue shift in the emission maxima (Fig. S22, ESI†). Further, these nanosheets facilitate efficient electron transfer between the HOMO and LUMO energy levels, thus **TfpBDH**-CONs exhibit enhanced luminescence with respect to the bulk COF. Considering these features, we selected **TfpBDH**-CONs only to study in detail of sensing behavior of the following different nitroaromatic analytes: TNP, TNT, DNP, DNT and NP. Solid **TfpBDH**-CONs were suspended in IPA. Upon excitation at 365 nm, this suspension displayed emission maxima at ∼447 nm which originates from the aromatic triphenyl chromophore units and a weak shoulder at ∼541 nm due to the charge transfer from the aromatic chromophore to the bisimide fragments. Luminescence spectrometric titration experiments were performed with above mentioned nitroaromatics (TNP, TNT, DNP, DNT and NP) at 5.4 × 10^–5^ (M) concentration in IPA. Fluorescence emission intensity was significantly quenched upon addition of increasing amounts of analyte solution. It was found that **TfpBDH**-CONs are highly sensitive and exhibit maximum *ca.* 63% quenching efficiency towards TNP at 5.4 × 10^–5^ (M) concentration in IPA over other nitroaromatic explosives (TNT: 31%, DNT: 3%, DNP: 23%, and NP: 4%) (Fig. 5b and S20, ESI†). The standard linear curve-fitting in the Stern–Volmer (SV) equation was employed to understand the quenching phenomena (Fig. 5c and S21, ESI†). The quenching constant for TNP was found to be 2.6 × 10^4^ M^–1^ and are in the order TNP ≫ TNT > DNP > NP > DNT. Although both TNP and TNT have the same standard reduction potential, the quenching efficiency of TNP is *ca.* 63% higher than that of TNT (31%) in the dispersed state. This result can be attributed to the electronic charge transfer between the picrate anion (TNP^–^) to the π-electronic cloud of the protonated **TfpBDH**-CONs. Furthermore, theoretical calculations (Fig. S27, ESI†) indicate that the ground-state electronic charge transfer takes place from the HOMO of picrate anion (TNP^–^) to the LUMO of the protonated **TfpBDH**-CONs and as a result, luminescence quenching of **TfpBDH**-CONs was observed. On the other hand, the HOMO energy level (–7.749 eV) of TNT is much lower than the LUMO energy level (–4.861 eV) of the **TfpBDH**-CONs. Therefore in this case, the electron transfer is not favorable and no such quenching phenomenon occurs (Fig. S28 and S29, ESI†). The photo-induced energy transfer from TNP^–^ to **TfpBDH**-CONs was further supported by fluorescence decay studies. Fluorescence decay was collected at 448 nm with an excitation wavelength of 365 nm in IPA at 25 °C. The fluorescence decay profile of **TfpBDH**-CONs (1 mg in 5 mL of IPA) at *λ*_ex_ = 390 nm exhibited biexponential decay with a lifetime of 0.75 ns, which reduced further to 0.35 ns in the presence of TNP (5.4 × 10^–5^ M). Reduction in the average lifetime upon addition of TNP indicates efficient energy transfer. The lifetime reduced sharply with increasing TNP concentration, which clearly suggests that the quenching is dynamic in nature (Fig. 5d). Further, we have investigated the change in thicknesses of CONs with respect to sonication time (samples collected at 0, 20, 30 and 60 minutes respectively). As expected, we found that the thickness of these CONs decreases gradually with aforementioned sonication time and monitored *via* AFM imaging with corresponding height profiles at (Fig. S16, ESI†). We have also attempted to understand the change in florescence intensity of these CONs recovered with different sonication time intervals (0, 20, 30 and 60 minutes).

It was observed that with increase in the sonication time, CONs exhibits a stronger PL peak almost ∼90 times intense compared to that of bulk COF samples (Fig. S24a, ESI†). Similarly, a notable effect was also seen in the UV excitation wavelength, gradual and obvious blue shift in excitation wavelength which varies significantly with CONs recovered at higher sonication time (60 minutes) that could be due to the decrease of stacking layers (Fig. S24b, ESI†). Further, curiously we have also monitored the effect of size using dynamic light scattering (DLS) experiment by collecting CONs samples at different time intervals (0, 20, 30 and 60 minutes respectively). It has been found from the DLS experiment that the average size of the CONs decreases as the sonication time increases (Fig. S31 and S32, ESI†). Such behavior has already been observed for other reported nanosheets.^5g,h^

**Fig. 6:**
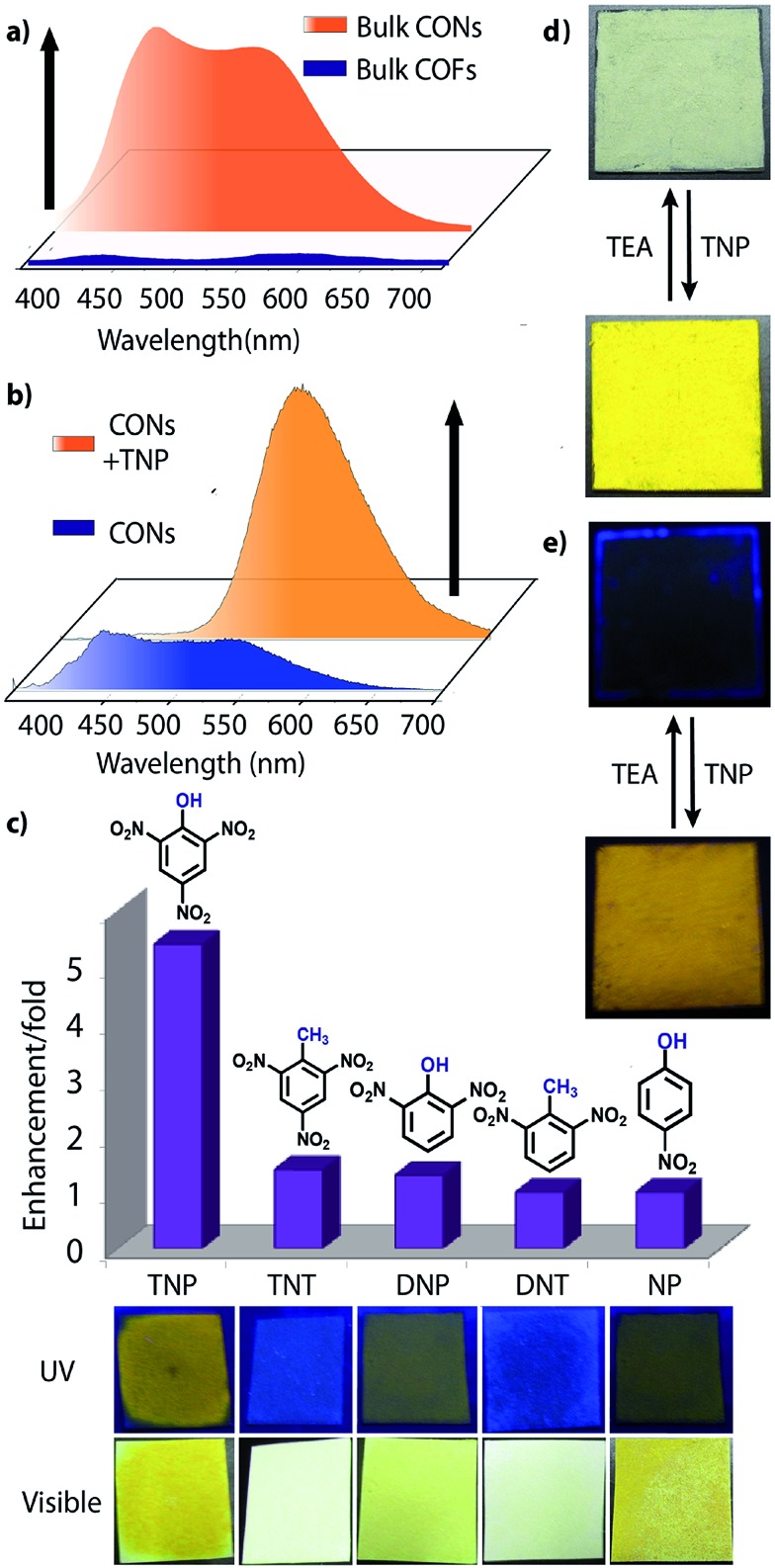
(a) Photoluminescent spectra of bulk **TfpBDH** and **TfpBDH**-CONs; (b) photoluminescent spectra showing turn on (PL enhancement) of bulk **TfpBDH**-CONs and with TNP; (c) photoluminescence enhancement plots of different analytes [at 5.4 × 10^–3^ (M)] with **TfpBDH**-CONs; (d and e) solid state sensing (turn on) by **TfpBDH**-CONs in presence of TNP under visible and UV light (365 nm) which also exhibits the reversible color change from blue to yellow and *vice versa* upon TEA treatment.

Besides solution phase detection, solid state luminescence experiments were also performed with **TfpBDH**-CONs in the presence of the same nitroaromatic analytes mentioned above. For this purpose, CONs-coated paper strips were used, which served as a simple, effective, fast, and low-cost support for detection of nitroaromatic analytes. Solid **TfpBDH**-CONs powder (10 mg in 50 mL of IPA) was deposited on the paper strip and displayed weak blue fluorescence under an UV lamp at an excitation wavelength of 365 nm. When only 200 μL [1 × 10^–3^ (M)] of TNP solution in IPA was deposited on the paper strip surface, a distinct colour change from blue to bright-yellow was immediately observed (Fig. 6c). The quantitative fluorescence spectral changes of the **TfpBDH**-CONs were also monitored in the solid state, by varying the TNP concentrations in IPA (0 to 1 × 10^–3^ M). Interestingly, the PL emission maxima in solid state (Fig. 6b) get red shifted and the intensity is enhanced (10 times higher than that of the pristine **TfpBDH**-CONs), with increasing TNP concentration (Fig. S23, ESI†). However, other nitroaromatic analytes did not show any significant effects on **TfpBDH**-CONs in the solid state.^10*a*^ This “turn on” sensing behaviour in the solid state could be due to the proton transfer from TNP to the basic nitrogen atom of the imine (–C=N) bond. We speculate that the protonation occurs predominantly on the surface of the CONs layers as these **TfpBDH**-CONs are being aggregated in the solid state. In order to understand the interaction between the acidic phenolic (–OH) groups of TNP with the imine bonds of **TfpBDH**-CONs and to check the reversibility of chemosensing in the solid state, we performed a simple experiment using the concept of acid–base interaction. In the presence of amine (TEA) vapour, the yellow colored paper strip of **TfpBDH**-CONs returns back to its initial gray colour within a few seconds (Fig. 6d and e). Such reversible protonation–deprotonation of the –C=N bond of the imine linkage with the phenolic –OH group is known for other imine bonded macrocyclic and polyazomethine compounds.^10*a*^ The above observations suggest that the CONs based chemosensor is highly selective, sensitive and reversible towards TNP over other explosives of the same family.^10*b*^

## Conclusions

In summary, we have successfully synthesized two imide based covalent organic frameworks by using simple Schiff-base reactions. These COFs are crystalline, porous and showed good hydrolytic and acidic stability. Thin layered covalent organic nanosheets were derived from these bulk COF materials *via* the liquid phase exfoliation method. Quite surprisingly, **TfpBDH**-CONs showed superior luminescent property under UV light, whereas the **TpBDH**-CONs are almost non-emissive. **TfpBDH**-CONs exhibit chemical sensing of nitroaromatic analytes, showing highly selective luminescence in both “turn on” (∼10 fold) and “turn off” (63%) modes. In the bulk aggregate state, the **TfpBDH**-CONs interact with the TNP molecule and show reversible “turn-on” sensing maximum lower concentration up to [1 × 10^–3^ (M)], which is also visually detectable by the naked eye. Once dispersed, **TfpBDH**-CONs showed luminescent quenching behavior under the influence of the same analytes. To the best of our knowledge, this is the first report where 2D-CONs have been engaged for fast and highly selective detection of nitroaromatic analytes *via* both turn-on/off sensing mechanisms. Although the quick and selective detection of TNT is highly desirable than any other nitroaromatics but we believe that the methodology presented in this work will open up new opportunities for the design and synthesis of two dimensional analytical platforms for the detection of highly explosive compounds like TNT in near future.
