# Supplementary material for: Chemical sensing in two dimensional porous covalent organic nanosheets
Source: Chem Sci. 2015 Apr 29;6(7):3931–9. doi: 10.1039/c5sc00512d (PMC5707461; doi:10.1039/c5sc00512d)
Supplement: Supplementary file 1 [file SC-006-C5SC00512D-s001.pdf]

Electronic Supplementary Material (ESI) for Chemical Science.
This journal is © The Royal Society of Chemistry 2015

Electronic Supporting Information

Chemical Sensing in Two Dimensional Porous Covalent Organic Nanosheets

Gobinda Das,[a,†] Bishnu P. Biswal,[a,d,†] Sharath Kandambeth,[a,d,†] V. Venkatesh,[b] Gagandeep Kaur,[b] Matthew Addicoat,[c] Thomas Heine,[c] Sandeep Verma[b] and Rahul Banerjee[a,d]*

[a]*Physical/Materials Chemistry Division, CSIR- National Chemical Laboratory, Dr. Homi Bhabha Road, Pune 411008, India.*

[b]*DST Thematic Unit of Excellence on Soft Nanofabrication, Indian Institute of Technology Kanpur, Kanpur-208016 (UP), India.*

[c]*Center for Functional Nanomaterials, School of Engineering and Science, Jacobs University Bremen, Bremen-28759, Germany.*

[d]*Academy of Scientific and Innovative Research (AcSIR), New Delhi, India*

Table of Contents

Section	Detail	Page No.
S-1	General Information	S3
S-2	Synthetic Procedures	S4-S6
S-3	Structural Modeling and Atomic Coordinates of COFs.	S6-S12
S-4	Powder X-Ray Diffraction Analysis (PXRD)	S12-S14
S-5	FT-IR Spectra	S14-S15
S-6	^{13}C CP/MAS NMR Spectra	S15-S16
S-7	Pore Size Distribution of TpBDH and TfpBDH	S16
S-8	Thermo Gravimetric Analysis (TGA)	S17
S-9	Scanning Electron Microscopy (SEM)	S17
S-10	High Resolution Transmission Electron Micrograph (HR-TEM) of COFs and CONs	S18-S19
S-11	AFM Images	S20
S-12	Chemical Stability Test (FT-IR)	S23
S-13	Photo-physical Studies and Theoritical Calculations	S24-S29
S-14	Tyndall effect	S30
S-15	Dynamic Light Scattering (DLS)	S31
S-16	References	S32

Section S-1: General Information

General Remarks: 1,3,5-Triformylphloroglucinol[1], pyromellitic-*N*,*N'*-bisaminoimide[2] and 1,3,5-tris(4-formylphenyl)benzene[3] were prepared by using literature procedures. All other reagents and solvents were commercially available and used as received. Powder X-ray diffraction (PXRD) patterns were recorded on a Rigaku, MicroMax-007HF with high intensity Microfocus rotating anode X-ray generator. All the samples were recorded in the (2 theta) range of 2–40 degrees and data was collected with the help of Control Win software. A Rigaku, R-axis IV++ detector was employed in wide-angle experiments. The radiation used was CuK (1.54 Å) with a Ni filter, and the data collection was carried out using an Aluminum holder. Fourier transform infrared (FT-IR) spectra were taken on a Bruker Optics ALPHA-E spectrometer with a universal Zn-Se ATR (attenuated total reflection) accessory in the 600-4000 cm^{-1} region or using a Diamond ATR (Golden Gate). Thermogravimetric analyses (TGA) were carried out on a TG50 analyzer (Mettler-Toledo) or a SDT Q600 TG-DTA analyzer under N_2 atmosphere at a heating rate of 10 ºC min^{-1} within a temperature range of 30-800 °C. SEM images were obtained with a Zeiss DSM 950 Scanning Electron Microscope and FEI, QUANTA 200 3D Scanning Electron Microscope with tungsten filament as electron source operated at 10 kV. The samples were sputtered with Au (nano-sized film) prior to imaging by a SCD 040 Balzers Union. HR-TEM images were recorded using FEI Tecnai G2 F30 X-TWIN TEM at an accelerating voltage of 300 kV. The HR-TEM samples were prepared by dropcasting the sample from isopropyl alcohol (IPA) on copper grids TEM Window (TED PELLA, INC. 200 mesh). All gas adsorption experiments (up to 1 bar) were performed on a *Quantachrome Quadrasorb* automatic volumetric instrument. Solid state NMR (SS-NMR) was taken in a Bruker 300 MHz NMR spectrometer. Both the samples were imaged using Agilent Technologies Atomic Force Microscopy (AFM) operating under contact mode, the sample was mounted on the XY stage of the AFM and the integral video camera (NAVITAR, Model N9451A-USO6310233 with the Fiber-lite source, MI-150 high intensity illuminator from Dolan-Jenner Industries was used to isolate the marked regions imbedded with the microscope. Micro fabricated silicon nitride cantilevers (PPP-CONT-20 from Nan sensors). The scanner model N9524AUSO7480132.xml/N9520A-USO7480152.xml was calibrated and used for imaging. The images were taken at room temperature in air with a scan speed of 2.8 lines/sec. Data acquisition and analysis was carried out using Pico View 1.8.2. The Images were processed by using Pico Image basic software. Operations such as leveling, filtering, line correction, and form removal were used

to processes the images for clarity. The third dimension of the layer assembly was determined by its 3D profile.

UV-DR spectra were recorded using JASCO V-570 spectrophotometer after dispersing the materials in solid state on a quartz plate. Steady-state fluorescence studies and time-resolved fluorescence lifetime measurements were performed using Horiba Jobin Yvon Fluorolog 3 spectrophotometer having a 450 W xenon lamp for steady-state fluorescence and nanoLED of 560 nm for fluorescence decay time. The tolerance range for each set temperature was maintained at 0.5 °C. For life time measurements, decay curves were obtained by the time-correlated single photon counting (TCSPC) technique.

Section S-2: Synthetic procedures

TpBDH: A Pyrex tube measuring (o.d. × i.d. = 10 × 8 mm^2 and length 18 cm) was charged with 1,3,5-triformylphloroglucinol (**Tp**) (210 mg, 0.3 mmol), pyromellitic-N,N$^{/}$-bisaminoimide (**BDH**) (110 mg, 0.45 mmol), 3 mL of *N*,*N*$^{/}$-dimethylacetamide, 1.5 mL of 1,4-dioxane and 0.5 mL of 6 M aqueous acetic acid. This mixture was sonicated for 10 minutes in order to get a homogenous dispersion. The tube was then flash frozen at 77 K (liquid N_2 bath) and degassed by three freeze-pump-thaw cycles. The tube was sealed off and then heated at 120 °C for 3 days. A dark red precipitate was collected by filtration and washed with acetone (25 mL)/DMAc (25 mL) three times. The powder collected was then solvent exchanged with acetone and dried at 180 °C under vacuum for 12 h to get corresponding **TpBDH** COF in ca. 81% isolated yield. **FT-IR (powder):** 1583, 1447, 1273, 1183, 815 cm^{-1}.

TfpBDH: The **TfpBDH-**COF was synthesised with a mixture of 1,3,5-tris(4-formylphenyl)benzene (**Tfp**) (117 mg, 0.3 mmol) and pyromellitic-*N*,*N*$^{/}$-bisaminoimide (**BDH**) (110 mg, 0.45 mmol) in presence of 6 M acetic acid (0.5 mL) using 1,4-dioxane (1.5 mL) and *N,N*$^{/}$-dimethylacetamide (3 mL) (1 : 2) as solvent combination. This mixture was sonicated for 10 minutes in order to get a homogenous dispersion. The tube was then flash frozen at 77 K (liquid N_2 bath) and degassed by three freeze-pump-thaw cycles. The tube was sealed off and then heated at 120 °C for 3 days. A gray coloured precipitate was collected by filtration and washed with dry acetone (25 mL)/ethanol (25 mL)/DMAc (25 mL) three times. The powder collected was then solvent exchanged with acetone and dried at 180 °C under

vacuum for 12 h to get the corresponding **TfpBDH** COF in ca. 78% isolated yield. **FT-IR (powder):** 1613, 1700, 1604, 152, 1389, 1228, 812 cm^{-1}.

General procedure for the synthesis of TpBDH-CONs and TfpBDH-CONs: 50 mg of as-synthesized COFs (**TpBDH** or **TfpBDH)** was placed in a 50 mL conical flask containing isopropyl alcohol (IPA) and sonicated at room temperature for 60 minutes. The resulting suspension was centrifuged at 1000 rpm for 5 minutes. The concentration of the material transferred from the settled solids to the solution as a result of sonication, the residue obtained after complete evaporation of solvent as CONs in ~ 4 wt% isolated yield. The dry powdered samples of CONs were used as such for characterizations like PXRD, TGA, FT-IR etc. to ensure their structural stability after grinding. For HR-TEM and AFM imaging we used 1 mg of CONs in 10 mL of IPA, sonicated for 5 minutes and subsequently coated on the carbon-coated copper grid (TEM) and HOPG (AFM), and dried at room temperature prior to imaging.

Procedure for sensing of nitroaromatic compounds using TfpBDH-CONs (Dispersion medium):

Solution phase experiments were carried out by spectrofluoremetric titration method. The fluorescence titration experiments were performed by gradually increasing the nitroaromatic analytes concentration. We made a homogeneous dispersion of CONs (1 mg of CONs in 3 mL of IPA), upon addition of TNP (also checked for TNT, DNP, DNT and NP) at different concentration [0 to 5.4×10^{-5} (M)], the fluorescent intensity of the CONs gradually decreased with the increasing concentration.

Procedure for sensing of nitroaromatic compounds using TfpBDH-CONs (Solid state):

20 mg of CONs were freshly collected by ultracentrifugation and dried under vacuum for 1 h. We have checked the sensing ability of these isolated CONs by filter paper strip assay experiments. Wherein test strips were prepared by drop casting the homogeneous suspension of CONs (1 mg in 5 mL) on a strip of filter paper (2 cm x 2 cm) then dried under vacuum at room temperature to get the fluorescent paper sensor. Upon addition of 200 μL [1 mg in 100 mL, 5.4×10^{-5} (M)] of TNP in IPA onto the test strip, the color of the strip immediately changed from blue to yellow (under 365 nm, UV lamp), which can also be visually observed with the naked eye. We have also performed the same experiment with other nitroaromatic

compounds such as TNT, DNP, DNT and NP, but they did not show any pronounced sensing response except TNP.

Procedure for sensing of nitroaromatic compounds using TfpBDH-CONs (Vapour phase):

A glass vial (5 mL capacity) containing 5 mg of dispersed **TfpBDH-**CONs in 3 mL of IPA was inserted in to a bigger glass vial (30 mL capacity) containing solid TNP at the bottom. The large glass vial was sealed by septum and vacuumed for different time interval (0-60 min.). The fluorescent spectra were recorded at the specified time interval.

Section S-3: Structure Modeling and Atomic Coordinates of COFs.

Atomic positions and cell sizes of modeled COF layers were optimized using Self-Consistent Charge Density Functional Tight-Binding (SCC-DFTB) method. Stacking of layers are affected by the Coulomb repulsion between the partial atomic charges in adjacent layers. Hence, we performed Mulliken population analysis for the charges. The adjacent layers were shifted with respect to each other in different directions in order to avoid Coulomb repulsion from charges alike. Several possibilities were considered, however, the best was taken from comparison of simulated PXRD pattern with the experimental. Interlayer separation was also determined from the comparison of PXRD patterns. The fractional coordinates of **TpBDH** and **TfpBDH** is given in **Table S1, S2** and **S3**.

Table S1. Fractional atomic coordinates for the unit cell of TpBDH

**TpBDH (AA-Eclipsed)**			
**Triclinic - *P*1**<br>***a*** **= 29.9665 Å,** ***b*** **= 27.9609 Å,** ***c*** **= 3.3029 Å**<br>$\boldsymbol{\alpha}$ **= 90.4°,** $\boldsymbol{\beta}$ **= 88.8°,** $\boldsymbol{\gamma}$ **= 117.1°**			
O1	0.30097	0.56026	0
O2	0.69902	0.43973	0
O3	0.43964	0.74066	0
O4	0.56037	0.25933	0
O5	0.25926	0.69902	0
O6	0.74074	0.30099	0

O7	0.51406	0.63746	0
O8	0.48595	0.36254	0
O9	0.36257	0.87674	0
O10	0.63743	0.12328	0
O11	0.12332	0.48584	0
O12	0.87667	0.51414	0
O13	0.35735	0.47393	0
O14	0.64266	0.52607	0
O15	0.52601	0.88331	0
O16	0.47399	0.1167	0
O17	0.11656	0.64266	0
O18	0.88343	0.35733	0
N1	0.39528	0.58262	0
N2	0.60473	0.41738	0
N3	0.41732	0.81265	0
N4	0.58269	0.18736	0
N5	0.1873	0.60469	0
N6	0.81269	0.3953	0
N7	0.4281	0.56175	0
N8	0.57192	0.43825	0
N9	0.43823	0.86635	0
N10	0.56178	0.13367	0
N11	0.13362	0.57187	0
N12	0.86636	0.42811	0
C1	0.31599	0.60971	0
C2	0.684	0.39028	0
C3	0.3902	0.70627	0
C4	0.60981	0.29374	0
C5	0.29367	0.68397	0
C6	0.70633	0.31603	0
C7	0.4108	0.63435	0
C8	0.5892	0.36564	0
C9	0.3656	0.7765	0
C10	0.63441	0.22351	0
C11	0.2235	0.58916	0
C12	0.7765	0.41085	0

C13	0.48461	0.58925	0
C14	0.5154	0.41075	0
C15	0.41076	0.89543	0
C16	0.58924	0.10459	0
C17	0.1046	0.51534	0
C18	0.89539	0.48464	0
C19	0.49824	0.54626	0
C20	0.50177	0.45374	0
C21	0.45376	0.95202	0
C22	0.54624	0.04799	0
C23	0.048	0.50173	0
C24	0.95199	0.49826	0
C25	0.45112	0.49642	0
C26	0.54889	0.50357	0
C27	0.50356	0.95466	0
C28	0.49645	0.04536	0
C29	0.0453	0.54888	0
C30	0.95468	0.45111	0
C31	0.40501	0.50517	0
C32	0.595	0.49482	0
C33	0.49479	0.89978	0
C34	0.50522	0.10024	0
C35	0.10015	0.59498	0
C36	0.89983	0.40501	0
C37	0.37215	0.64962	0
C38	0.62785	0.35037	0
C39	0.3503	0.72256	0
C40	0.64971	0.27745	0
C41	0.2774	0.62779	0
C42	0.72259	0.37222	0
C43	0.54864	0.55148	0
C44	0.45137	0.44851	0
C45	0.44856	0.99723	0
C46	0.55145	0.00279	0
C47	0.00282	0.45133	0
C48	0.99717	0.54865	0

H1	0.45304	0.66404	0
H2	0.54696	0.33596	0
H3	0.33594	0.78911	0
H4	0.66407	0.2109	0
H5	0.21096	0.54691	0
H6	0.78904	0.4531	0
H7	0.35188	0.55701	0
H8	0.64814	0.44297	0
H9	0.44288	0.79475	0
H10	0.55713	0.20528	0
H11	0.2051	0.64811	0
H12	0.79486	0.35187	0
H13	0.58508	0.59013	0
H14	0.41494	0.40987	0
H15	0.40993	0.99509	0
H16	0.59008	0.00494	0
H17	0.00501	0.41487	0
H18	0.99496	0.58512	0

Table S2. Fractional atomic coordinates for the unit cell of TfpBDH

**TfpBDH (AA-Eclipsed)**			
**Hexagonal-*P6/m*** ***a* = *b* = 44.6 Å, *c* = 3.5 Å** ***α* = 90°, *β* = 90°, *γ* = 120°**			
H1	0.44163	0.81831	0
H2	0.4183	0.75722	0
H3	0.33923	0.80479	0
H4	0.31436	0.74353	0
H5	0.38906	0.86394	0
H6	0.39624	0.70478	0
H7	0.55677	0.55653	0
C1	0.46761	0.46778	0
C2	0.4691	0.49996	0
C3	0.4416	0.50871	0
C4	0.50036	0.53101	0
O1	0.41075	0.48778	0

N1	0.4549	0.54365	0
N2	0.4348	0.56001	0
C5	0.44731	0.59329	0
C6	0.36905	0.68831	0
C7	0.34783	0.70346	0
C8	0.81615	0.42394	0
C9	0.74272	0.37942	0
C10	0.80167	0.38796	0
C11	0.79414	0.43774	0
C12	0.76577	0.36611	0
C13	0.7582	0.41606	0
C14	0.49174	0.55847	0
O2	0.51261	0.58931	0

Table S3. Fractional atomic coordinates for the unit cell of TfpBDH

Change in unit cell parameters of TfpBDH (ABC-Staggered)	
**Before**	**After refinement**
**Trigonal, *R*3**<br>$a = b = 44.0018$ Å, $c = 6.6472$ Å<br>$\alpha = \beta = 90°$, $\gamma = 120°$	**Triclinic, *P*1**<br>$a = 44.0018$ Å, $b = 44.0218$ Å, $c = 6.6472$ Å<br>$\alpha = 89.7351°$, $\beta = 89.7327°$, $\gamma = 119.892°$

TfpBDH (ABC-Staggered)			
**Trigonal, *R*3**<br>$a = b = 44.0018$ Å, $c = 6.6472$ Å<br>$\alpha = \beta = 90°$, $\gamma = 120°$			
C1	0.47098	0.50266	0.58912
C2	0.44303	0.51194	0.6282
C3	0.49798	0.53036	0.47811
C4	0.42041	0.60469	0.45909
C5	0.43149	0.63697	0.36029
C6	0.41024	0.65207	0.3554
C7	0.36588	0.60312	0.54683
C8	0.38711	0.58797	0.55231
C9	0.37701	0.63556	0.44935

C10	0.35478	0.65214	0.44755
C11	0.47288	0.47287	0.64435
C12	0.44354	0.58965	0.45914
C13	0.48777	0.55772	0.44224
C14	0.31794	0.63151	0.44573
C15	0.53009	0.4999	0.47244
C16	0.55804	0.49061	0.43336
C17	0.50309	0.47219	0.58344
C18	0.58063	0.39783	0.60254
C19	0.56955	0.36556	0.70127
C20	0.59079	0.35044	0.70616
C21	0.63517	0.39941	0.51487
C22	0.61395	0.41457	0.50938
C23	0.62404	0.36697	0.61229
C24	0.64626	0.35039	0.61411
C25	0.5282	0.52969	0.4172
C26	0.55752	0.41289	0.60246
C27	0.51329	0.44483	0.61932
C28	0.6831	0.37105	0.61589
N1	0.4548	0.54508	0.53652
N2	0.43336	0.55928	0.5414
N3	0.54628	0.45748	0.52506
N4	0.56771	0.44326	0.5202
O1	0.49678	0.41737	0.71198
O2	0.41561	0.4955	0.71976
O3	0.50428	0.58517	0.34959
O4	0.58547	0.50705	0.34181

Table S4. Refinement data for the various stacking arrangements.

**TfpBDH (AA)**	Rwp = 3.04%	Rwp (w/o bck) = 20.55%	Rp = 1.73%
**TfpBDH (AB)**	Rwp = 9.56%	Rwp (w/o bck) = 14.94 %	Rp = 5.10%
**TfpBDH (ABC)**	Rwp = 3.08%	Rwp (w/o bck) = 3.85%	Rp = 2.57%

Table S5. Theoritical energy calculations of different stacking arrangments for TfpBDH.

	Inter layer distance (Å)	Total Energy (a.u.)	Stacking energy (kcal/mol)	HOMO-LUMO gap (eV)
**Monolayer**	-	-227.806096	-	1.714
**Eclipsed AA**	3.50	-455.937296	102.00	1.232
**Slipped AA (1)**	3.23	-455.767697	48.79	1.607
**Slipped AA (2)**	3.07	-455.756073	45.14	1.628
**Slipped AA (3)**	3.38	-455.972290	112.98	1.323
**Tilted AA**	3.57	-455.949640	105.88	1.332
**Staggered AB**	3.05	-455.7390962	39.82	1.512
**Staggered ABC**	2.22	-683.792234	78.22	1.689

Section S-4: Powder X-Ray Diffraction (PXRD) Analysis

In order to elucidate the structure of these COFs and to calculate the unit cell parameters, all possible 2-D models (AA/AB/ABC) were built using AuToGraFS and optimized by the Density Functional Tight-Binding method (SCC-DFTB). Several stacking possibilities were considered for reasons reported in the literature.[4] The experimental PXRD patterns are agreeable with the simulated patterns of some near-eclipsed stacking models of **TpBDH**. However, for **TfpBDH** it is closely macthing with the ABC stacked model and not with the AA-eclipsed or AB-staggered one. (Figure S1 and S2 in ESI). Hence, we propose structures close to triclinic space group (*P*1) for **TfpBDH** and **TpBDH** by comparing the experimental and simulated PXRD patterns. Refinements of PXRD pattern were done using Reflex module of Material studio.[5]

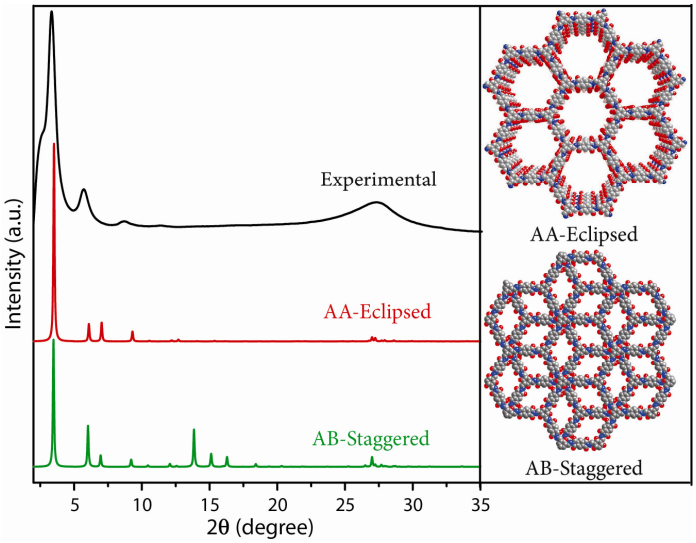

Figure S1: PXRD patterns of experimental **TpBDH** (black) compared with the AA-eclipsed (red) and AB-staggered (green) stacking models.

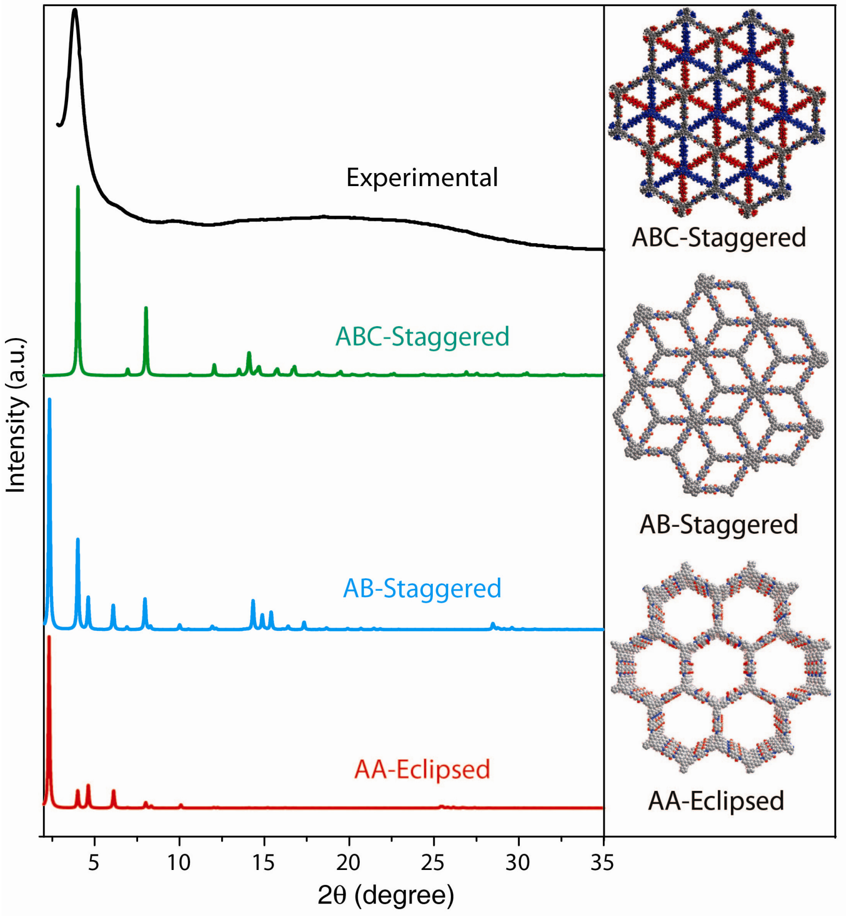

Figure S2: PXRD pattern of experimental **TfpBDH** (black) compared with the ABC-staggered (green), AB-staggered (cyan) and AA-eclipsed (red) stacking models.

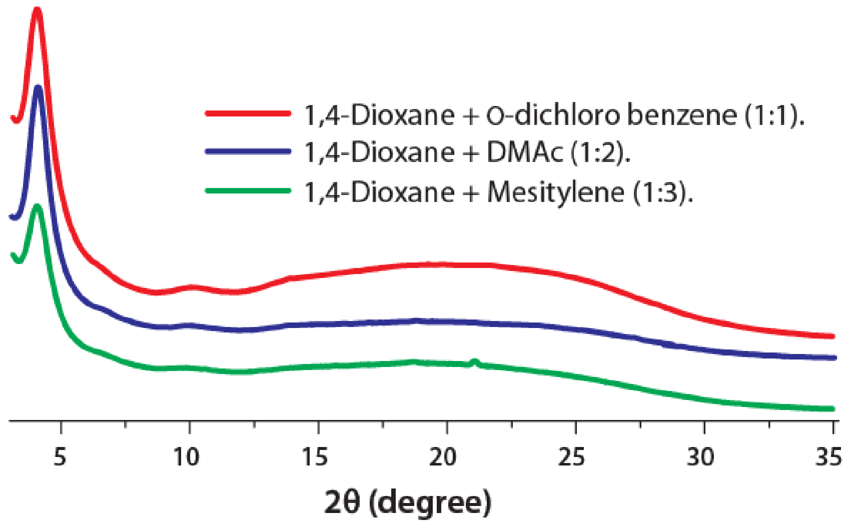

Figure S3: PXRD patterns of **TfpBDH** in different organic solvent combinations.

Section S-5: FT-IR

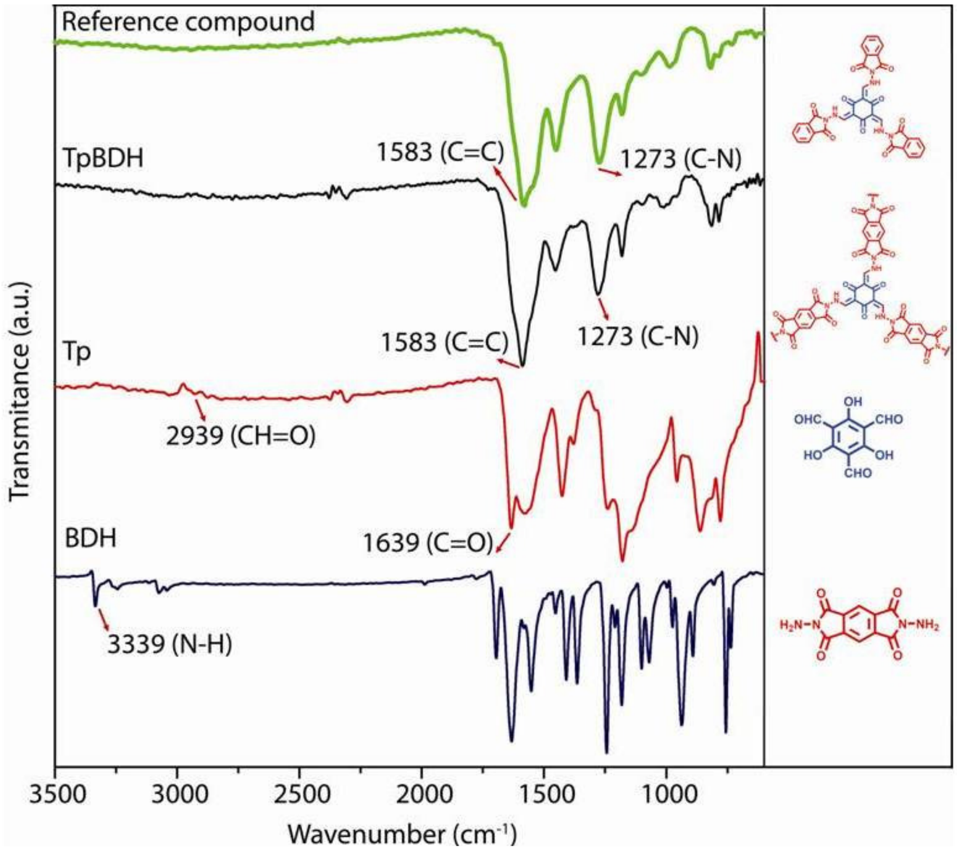

Figure S4: FT-IR spectra of **TpBDH** (Black) compared with the reference compound (green), 1,3,5-triformylphloroglucinol (**Tp**) (red), and pyromellitic-*N*,*N*′-bisaminoimide (**BDH**) (Blue).

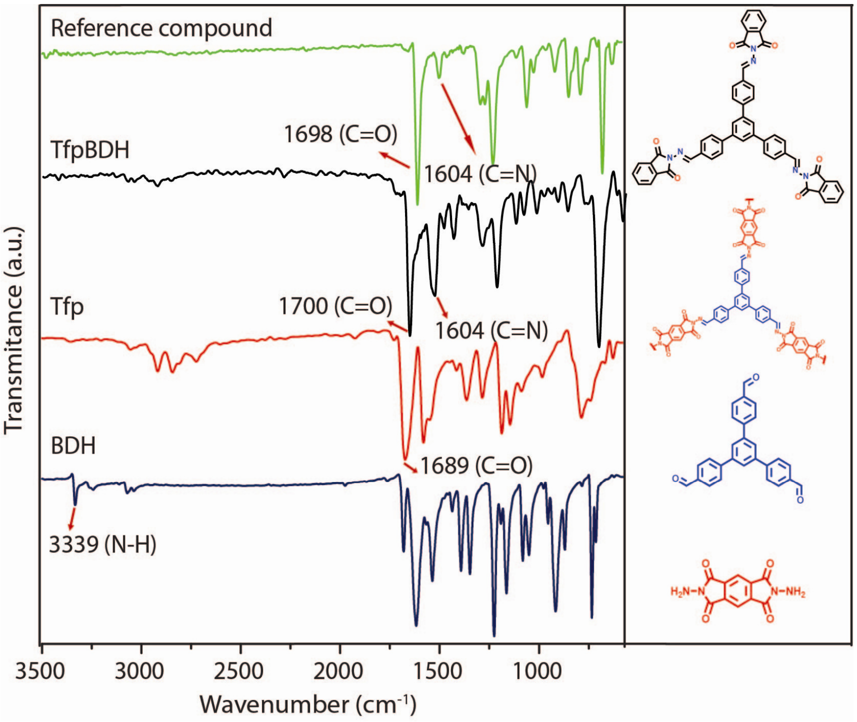

Figure S4a: FT-IR spectra of **TfpBDH** (black) compared with the reference compound (green), 1,3,5-triformylbenzene (**Tfp**) (red), and pyromellitic-*N*,*N*′-bisaminoimide (**BDH**) (Blue).

Section S-6: C^{13}-CP-MAS-Solid state-NMR

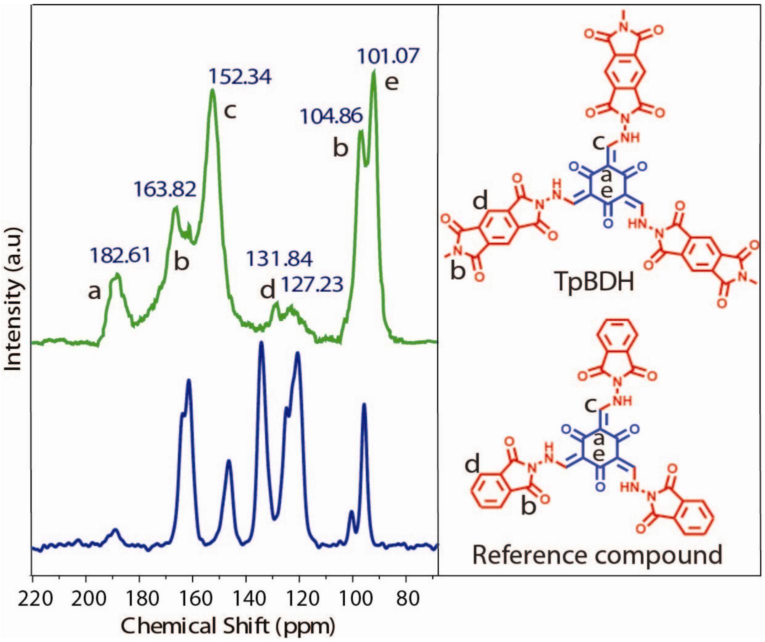

Figure S5: ^{13}C CP-MAS NMR spectrum of **TpBDH** (green) and reference compound (blue).

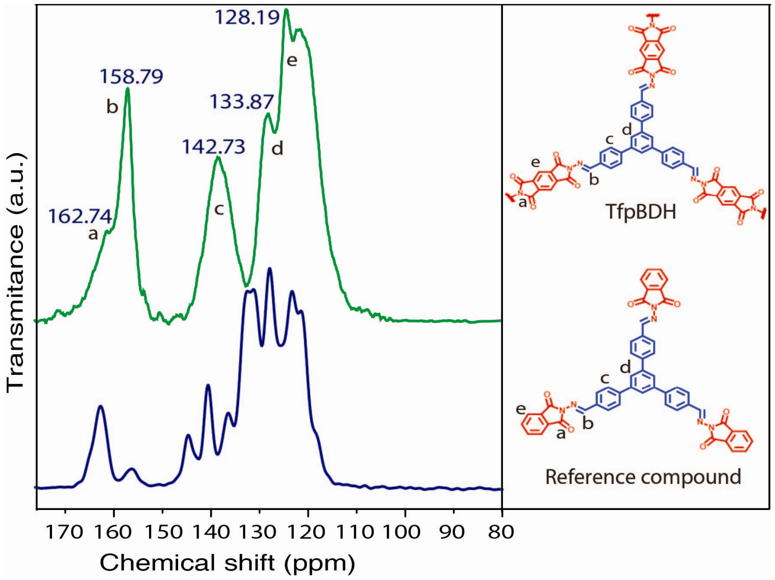

Figure S6: ^{13}C CP-MAS NMR spectrum of **TfpBDH** (green) and reference compound (blue).

Section S-7: <u>Pore size distribution of TpBDH and TfpBDH</u>

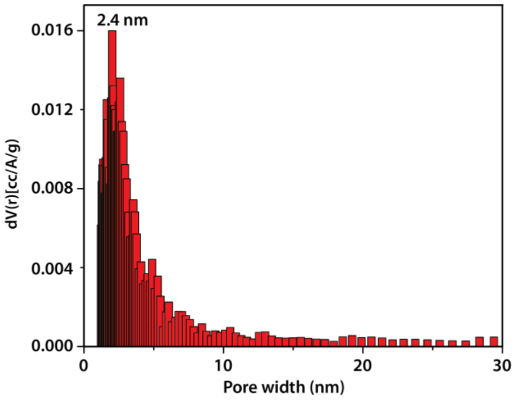

Figure S7: Pore size distribution plot of **TpBDH.**

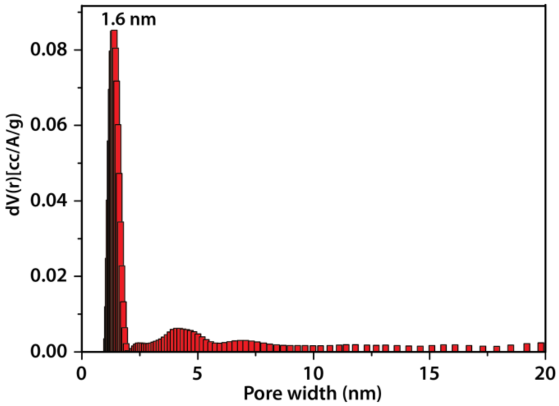

Figure S8: Pore size distribution plot of **TfpBDH**.

Section S-8: Thermo Gravimetric Analysis (TGA)

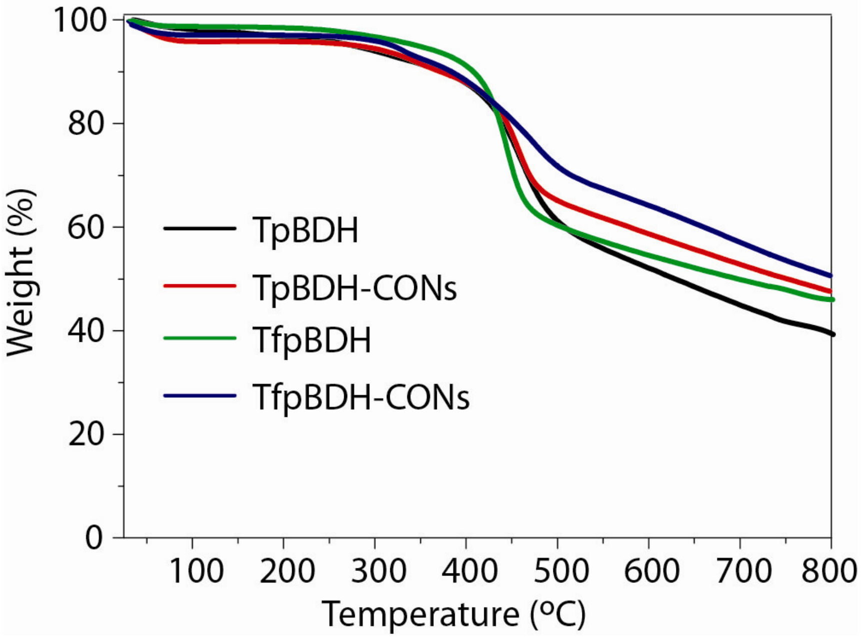

Figure S9: TGA profiles of activated **TpBDH**, **TfpBDH**, **TpBDH-**CONs and **TfpBDH-**CONs samples respectively under N_2 atmosphere.

Section S-9: Scanning Electron Microscopy (SEM)

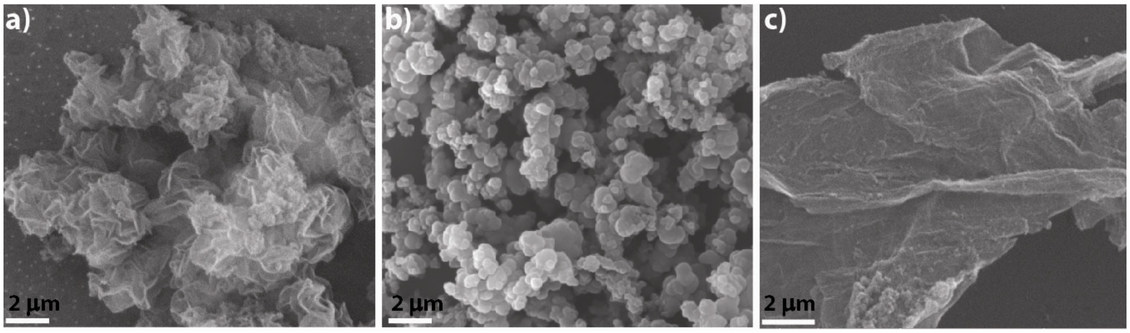

Figure S10: SEM images of a) **TpBDH**, b) **TfpBDH** and c) **TfpBDH**-CONs.

Section S-10: Transmission Electron Microscopy (TEM)

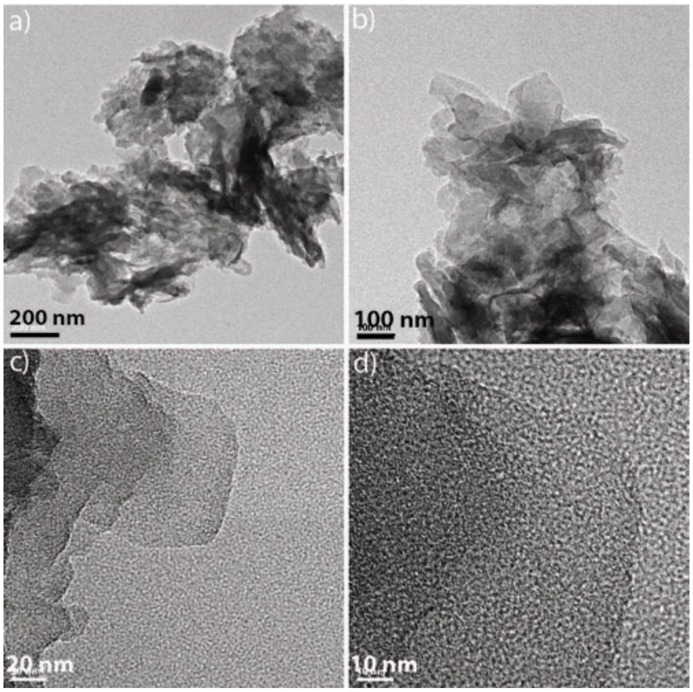

Figure S11: a-d) HR-TEM images of **TpBDH** at different magnifications.

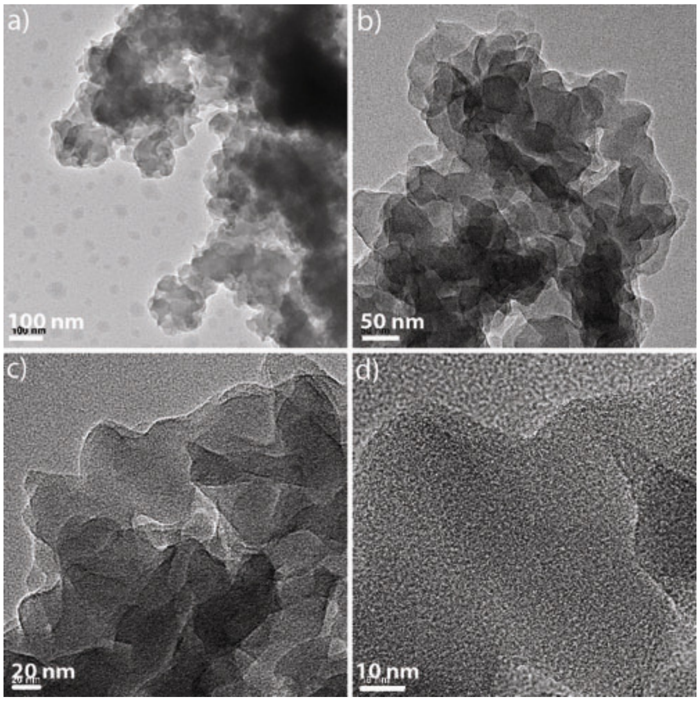

Figure S12: a-d) HR-TEM images of **TfpBDH** at different magnifications.

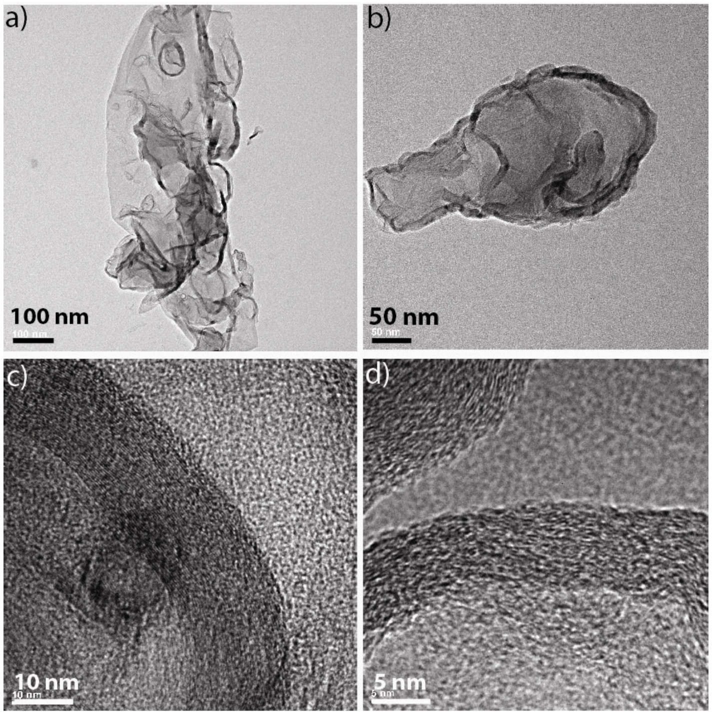

Figure S13: a-d) HR-TEM images of **TpBDH-**CONs with different magnifications.

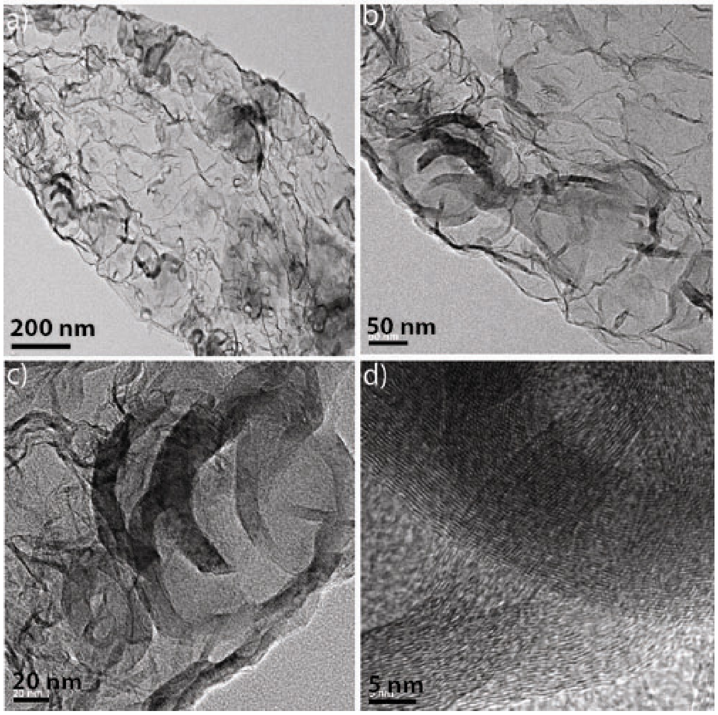

Figure S14: a-d) HR-TEM images of **TfpBDH-**CONs with different magnifications.

Section S-11: Atomic Force Microscopy (AFM)

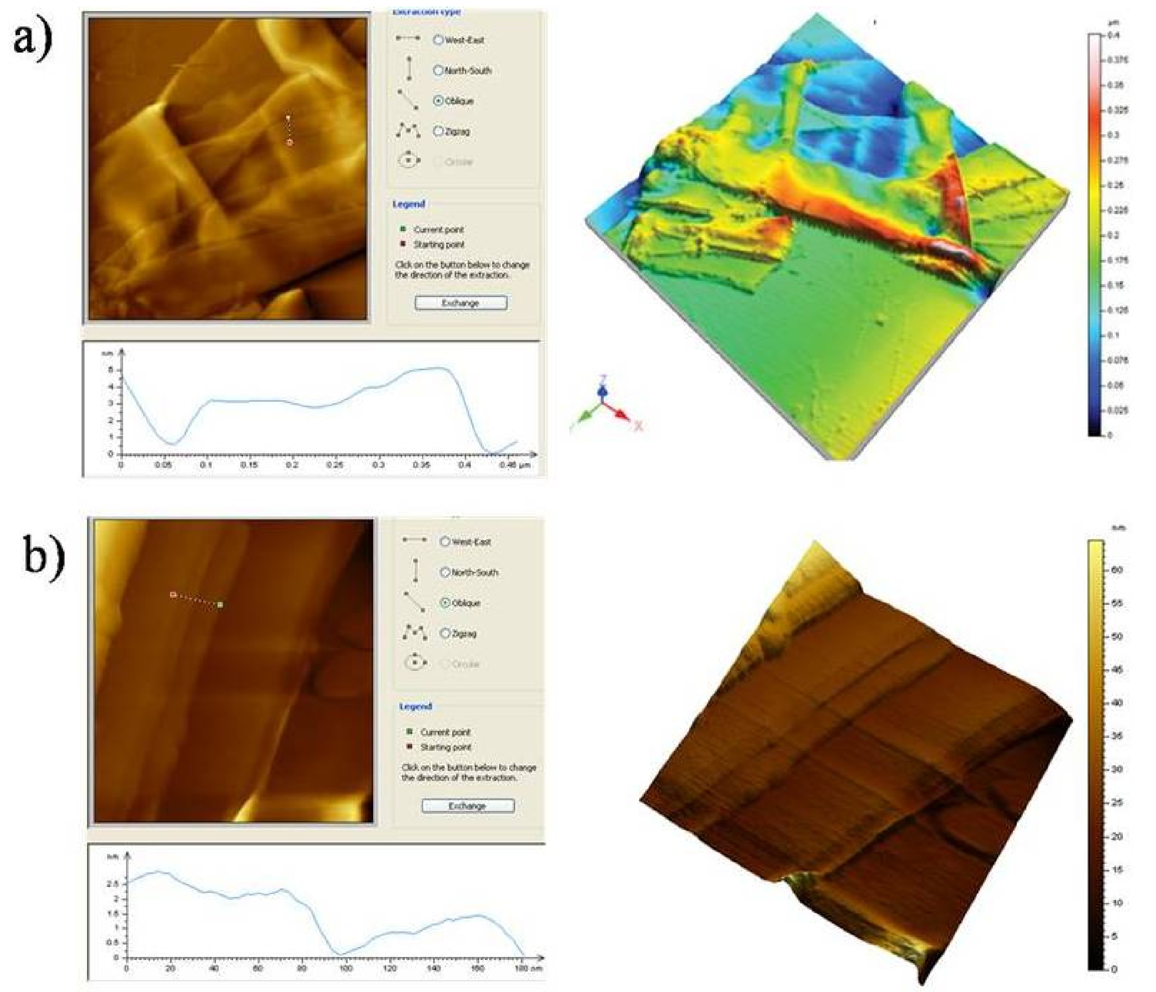

Figure S15: AFM images of a) **TfpBDH-**CONs and 3D-AFM view of **TfpBDH-**CONs; b) **TpBDH-**CONs and 3D-AFM view of **TpBDH-**CONs. (Note: AFM images of S15a and S15b also presented in the main paper).

Time (min)	AFM
0	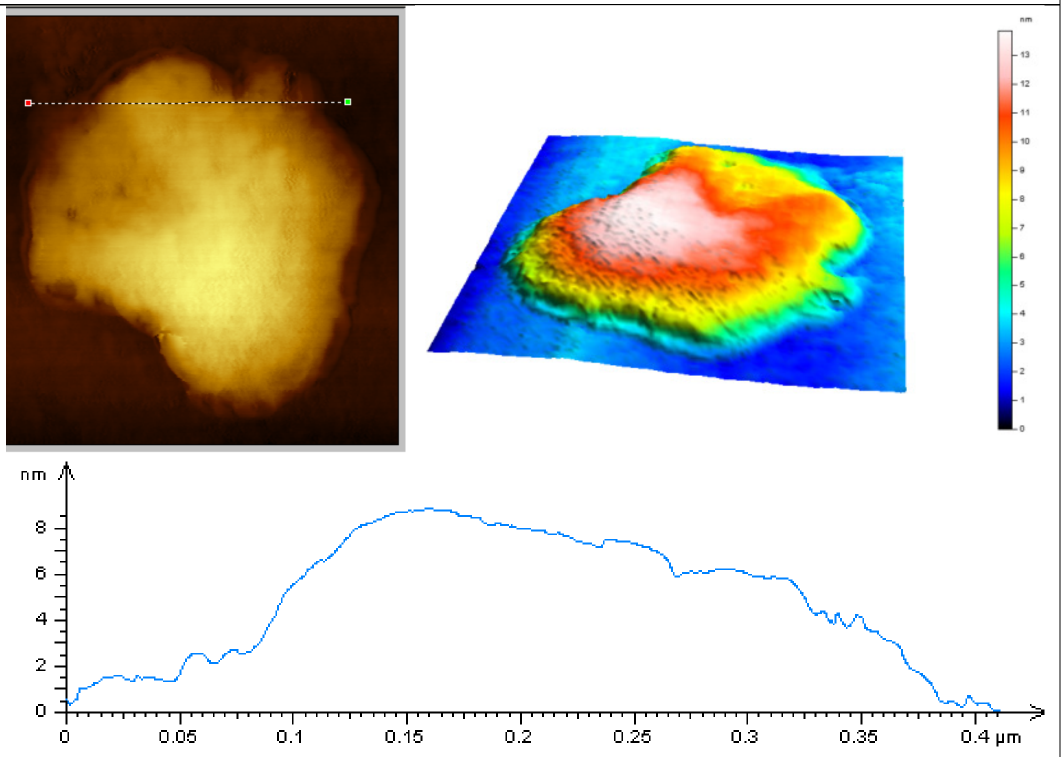
20	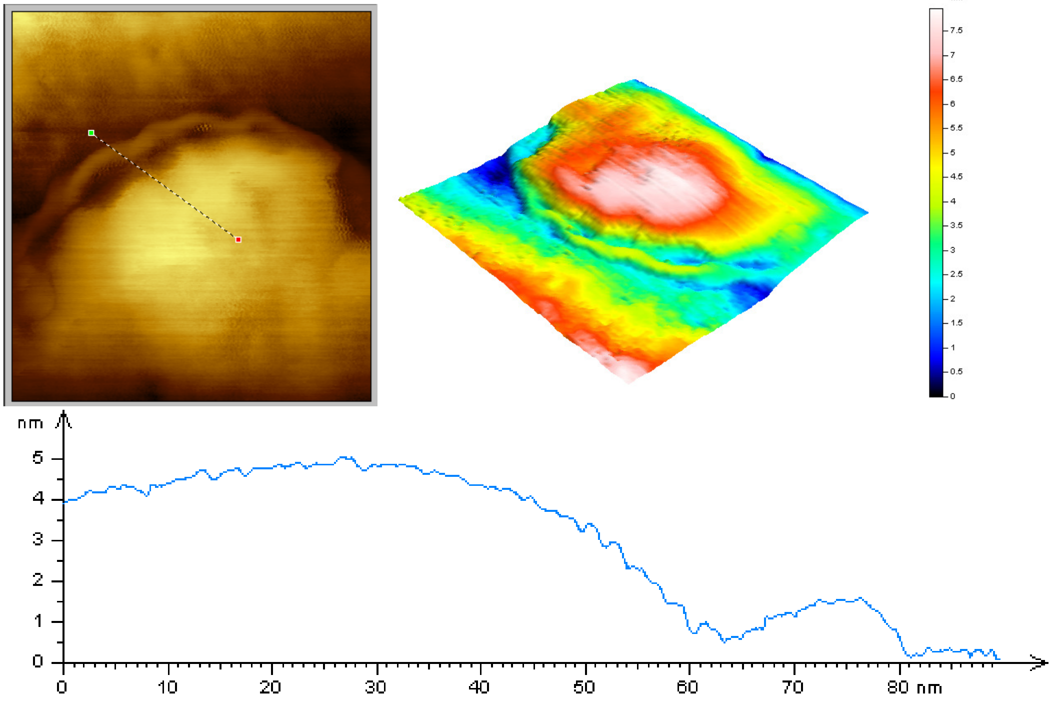 (Continue)

**30**	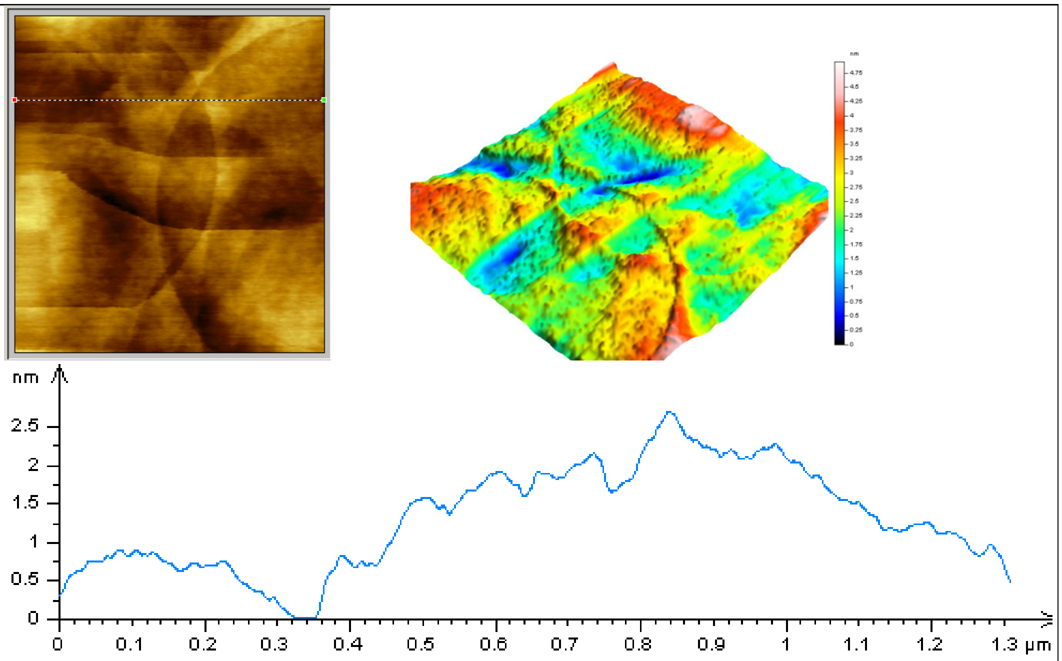
**45**	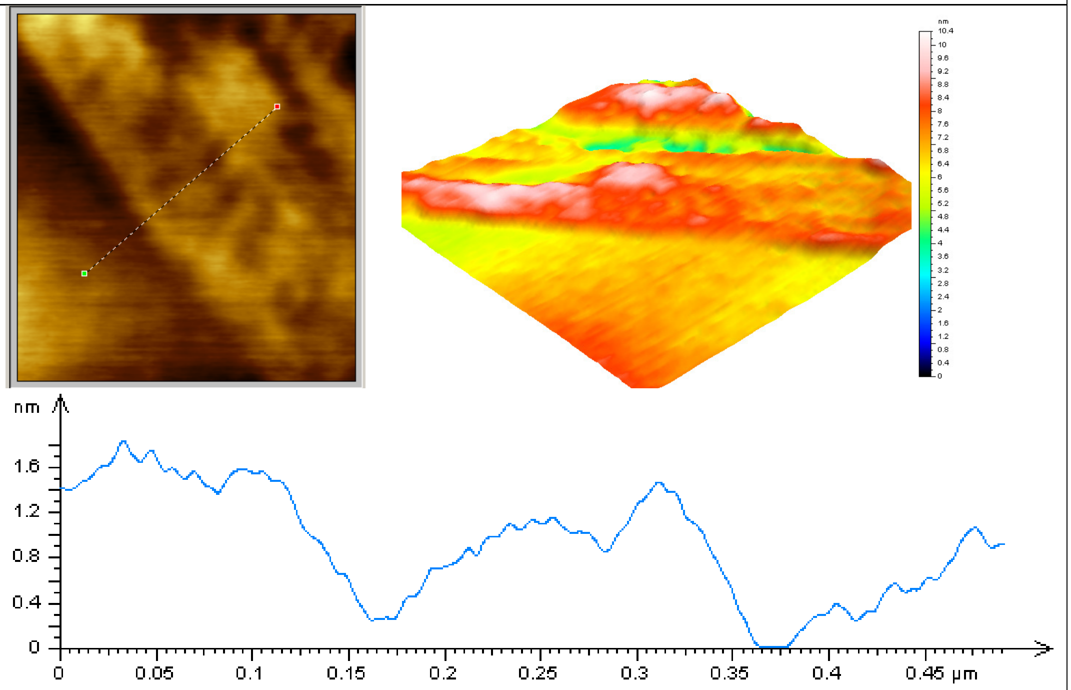<br>(Continue)

**60**	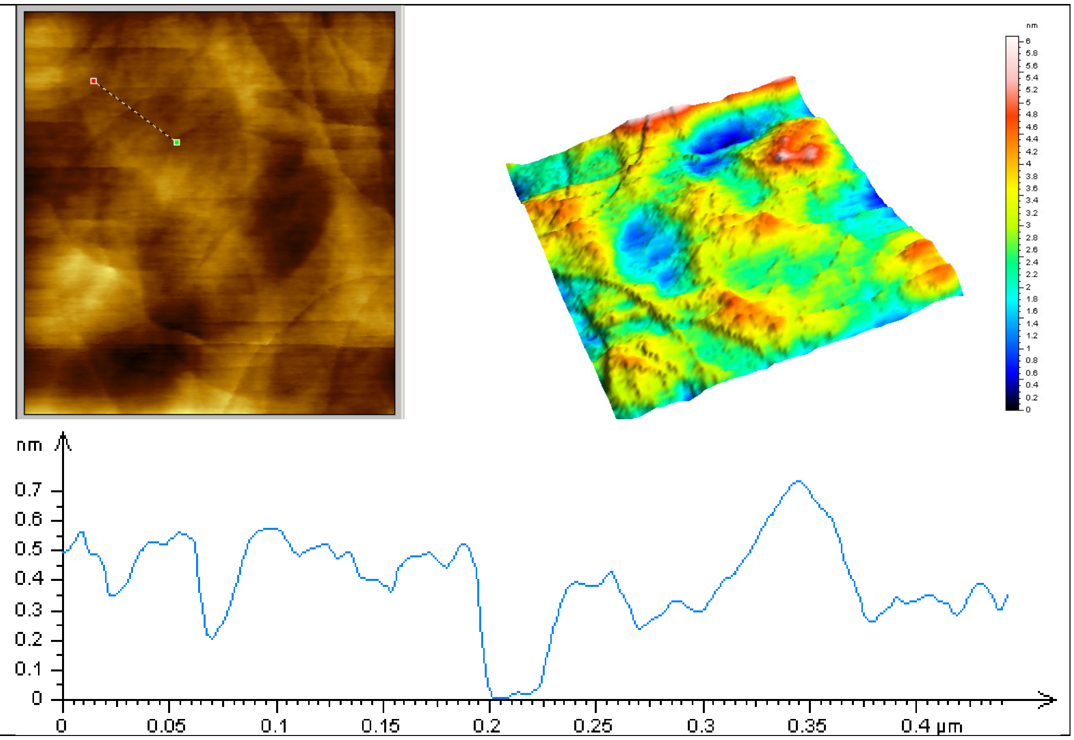

Figure S16: AFM images of **TfpBDH-**CONs recovered at different sonication time intervals.

Section S-12: Chemical Stability Tests (FT-IR)

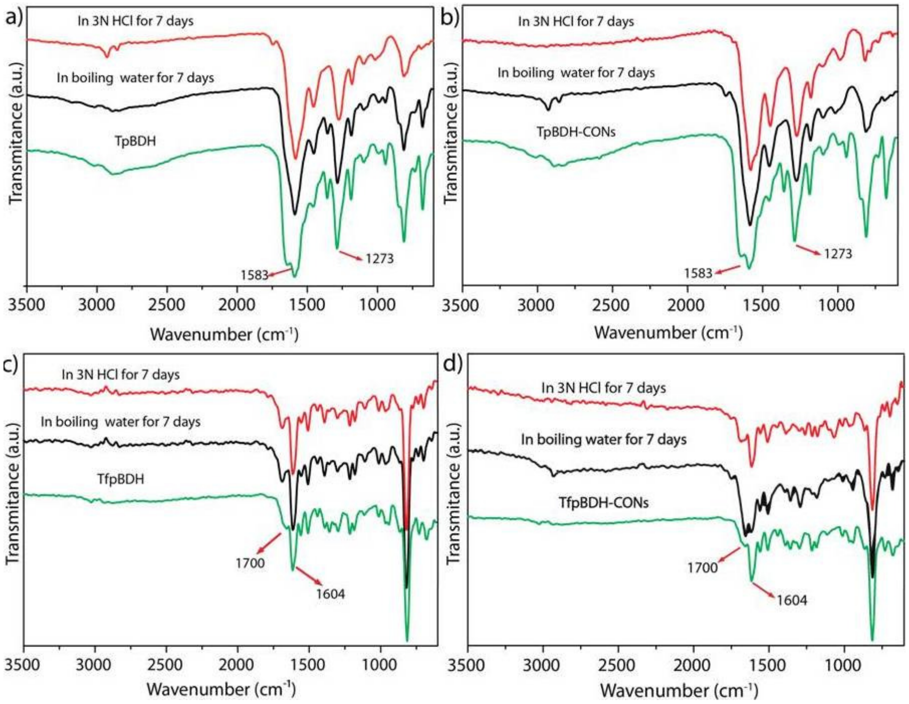

Figure S17: FT-IR spectra of a) **TpBDH**, b) **TpBDH-**CONs, c) **TfpBDH** and d) **TfpBDH-**CONs showing stability in 3 N HCl and in boiling water for 7 days.

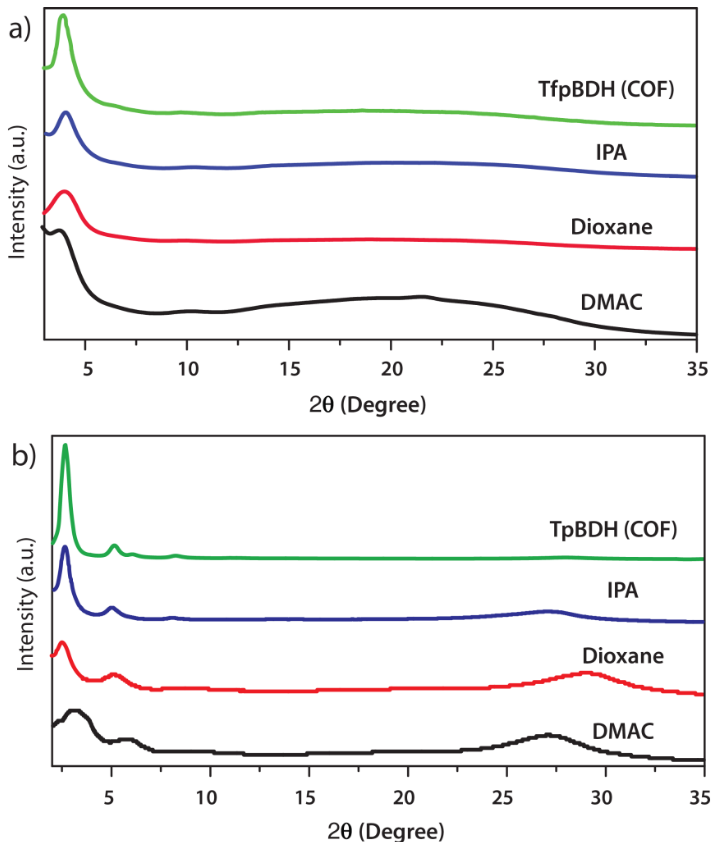

Figure S18: PXRD pattern of a) **TfpBDH**-CONs and b) **TpBDH**-CONs synthesized in diffrent organic solvents compared with parent **TfpBDH** (COF).

Section S-13: Photo-physical studies (Dispersion medium)

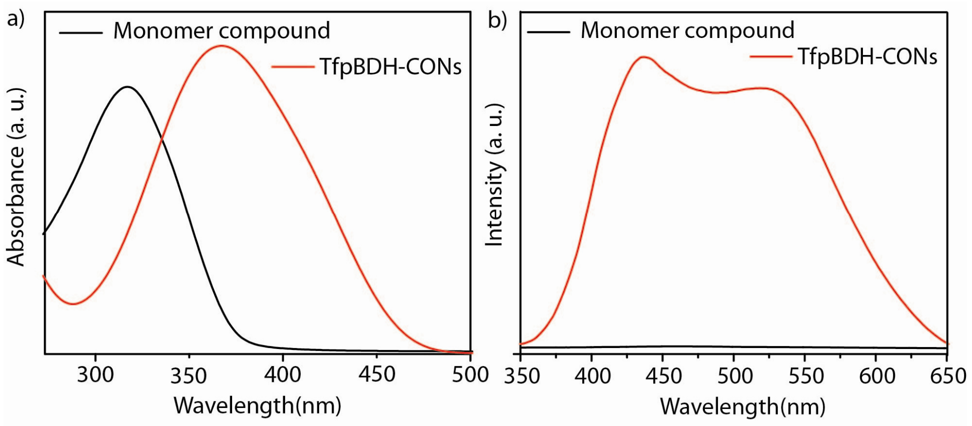

Figure S19: a) Normalized UV-visible spectra; b) fluorescence spectra of monomer compound (black) and **TfpBDH-**CONs (red).

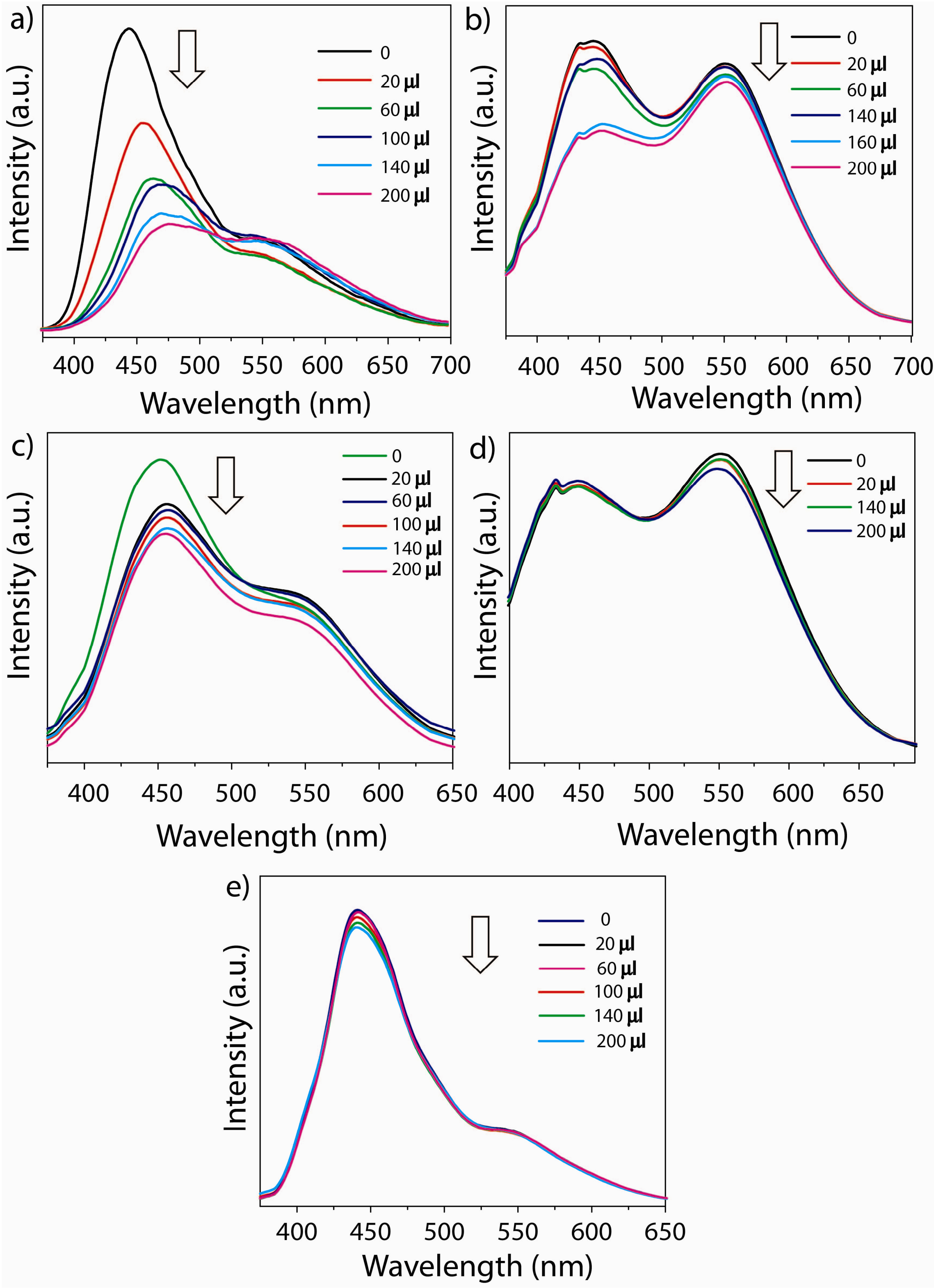

Figure S20: Fluorescence quenching of **TfpBDH-**CONs with (a) TNP, (b) TNT, (c) DNP, (d) NP and (e) DNT anlytes respectively.

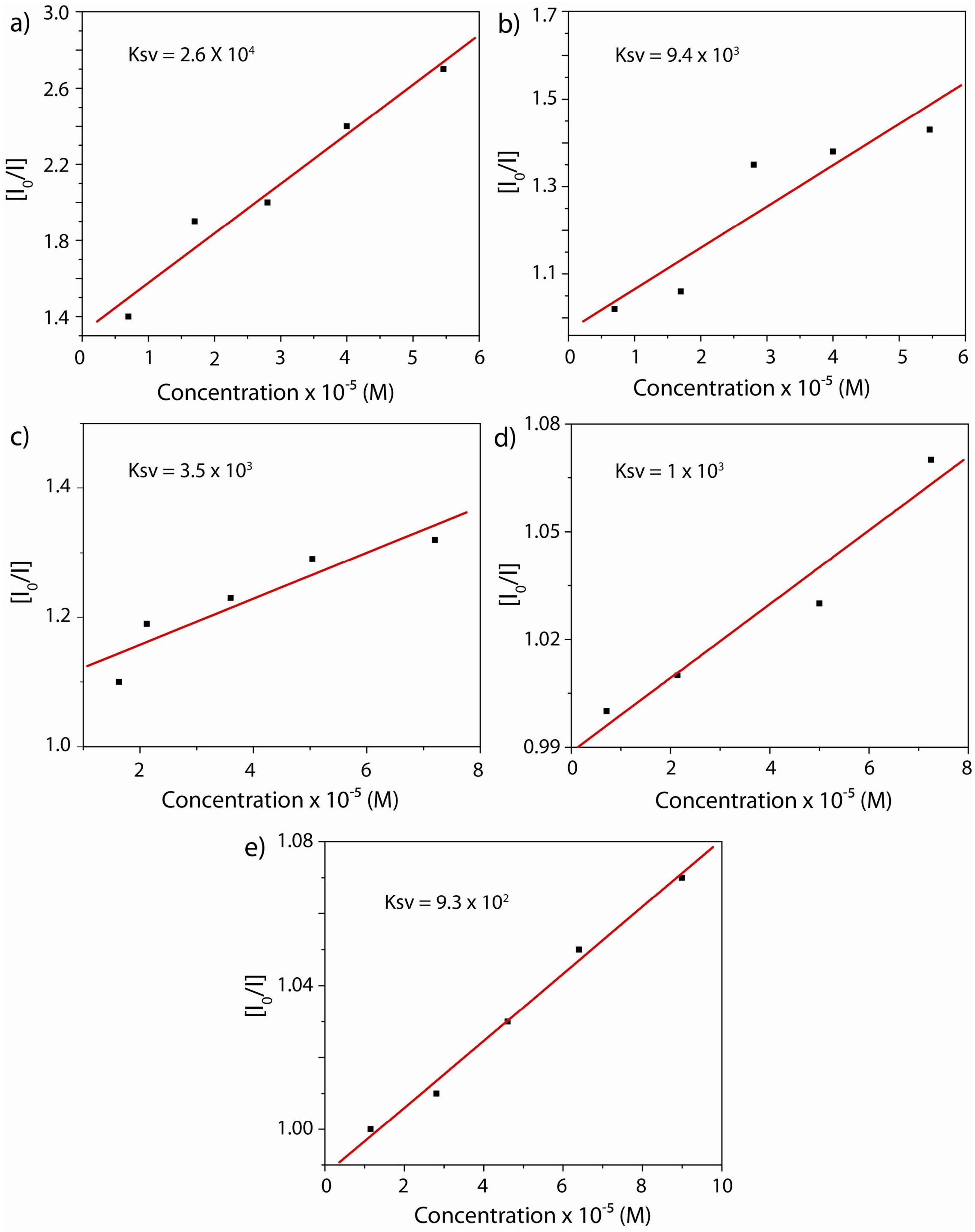

Figure S21: Stern-Volmer plots obtained from the titration of **TfpBDH-**CONs with (a) TNP, (b) TNT, (c) DNP, (d) NP and (e) DNT anlytes, respectively.

Photo-physical studies (Solid state)

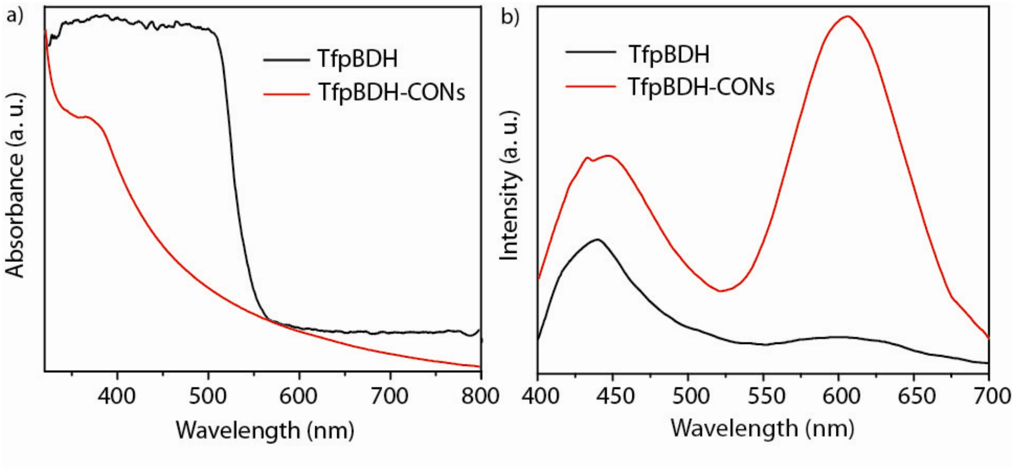

Figure S22: a) Normalized UV-visible spectra; b) fluorescence spectra of **TfpBDH** (black) and **TfpBDH-**CONs (red).

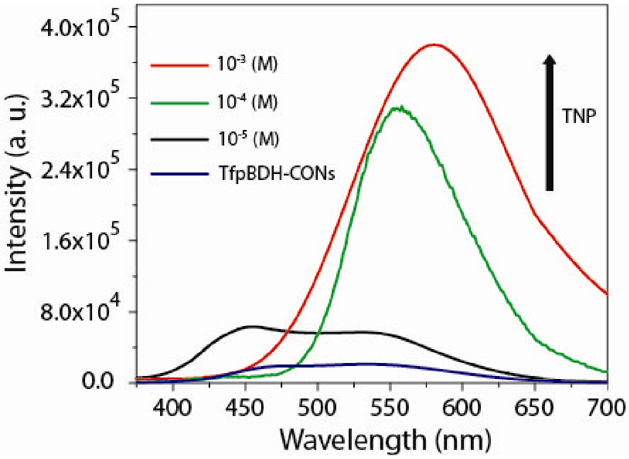

Figure S23: Fluorescence enhancement of **TfpBDH-**CONs by addition of TNP in IPA at different analyte concentrations.

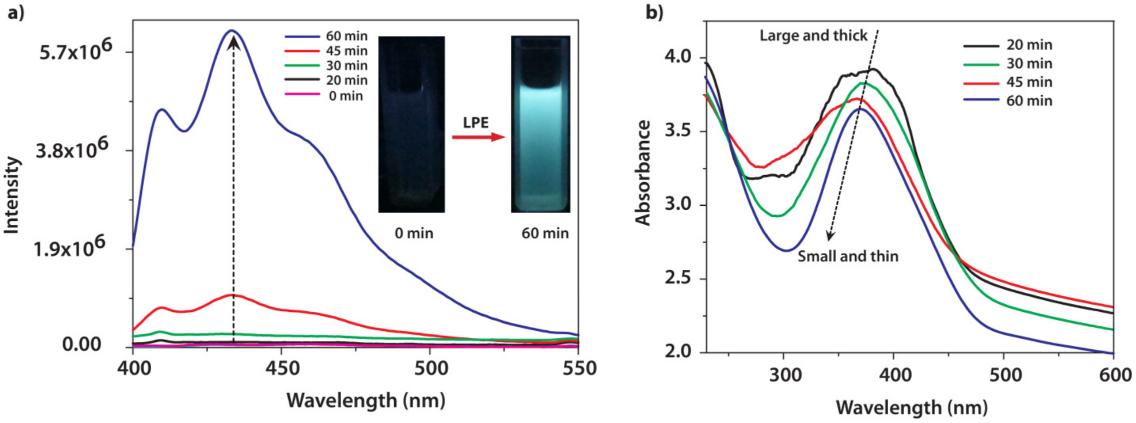

Figure S24: a), b) Luminescent/ UV-visible study at different sonication time intervals.

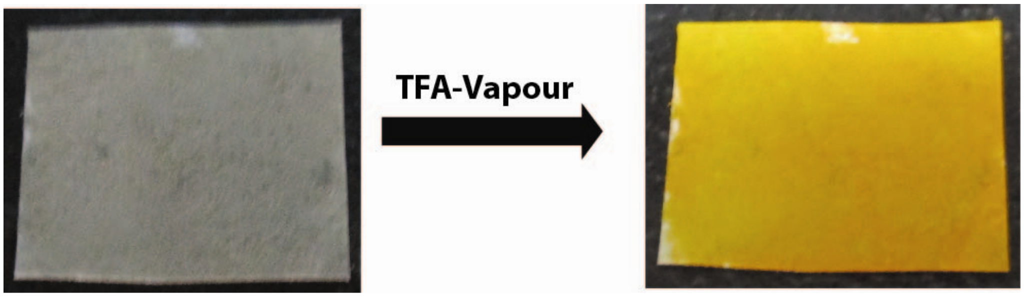

Figure S25: Digital photographs showing colour change of **TfpBDH-**CONs coated paper strip upon expose to trifloroacetic acid (TFA) vapour under visible light.

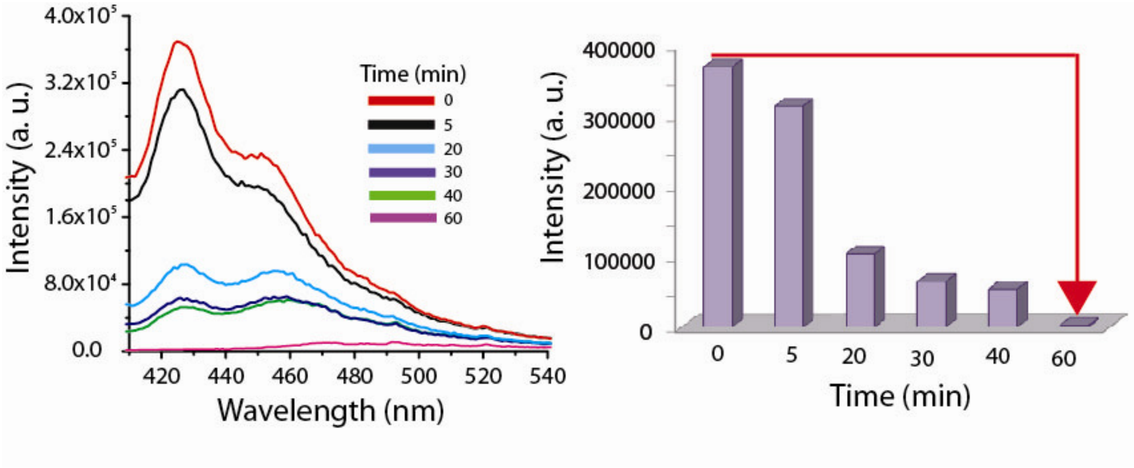

Figure S26: Fluorescence quenching of **TfpBDH-**CONs in IPA, in the presence of TNP vapour, at different time interval.

Theoritical calculations

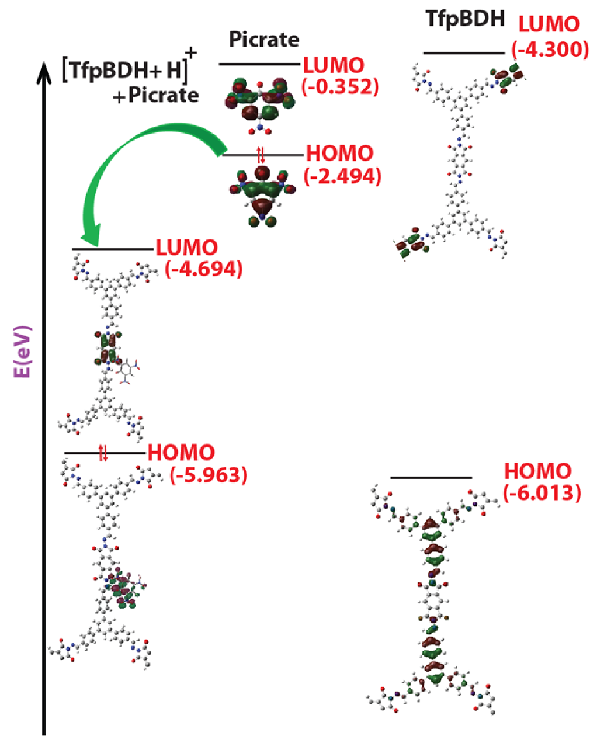

Figure S27: Pictorial representation of ground state charge transfer process occurring from HOMO of the picrate anion to the LUMO of the protonated **TfpBDH**-CONs**.**

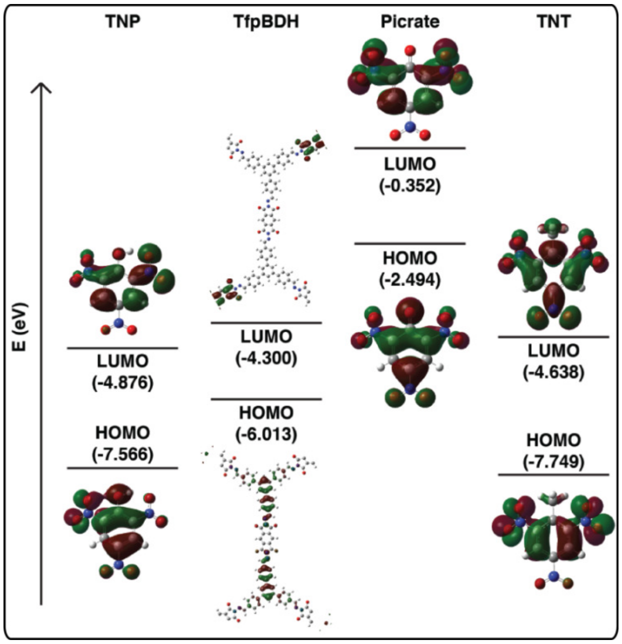

Figure S28: HOMO-LUMO energy profile of TNP, TfpBDH-CONs, TNP^{-} (picrate anion) and TNT respectively**.**

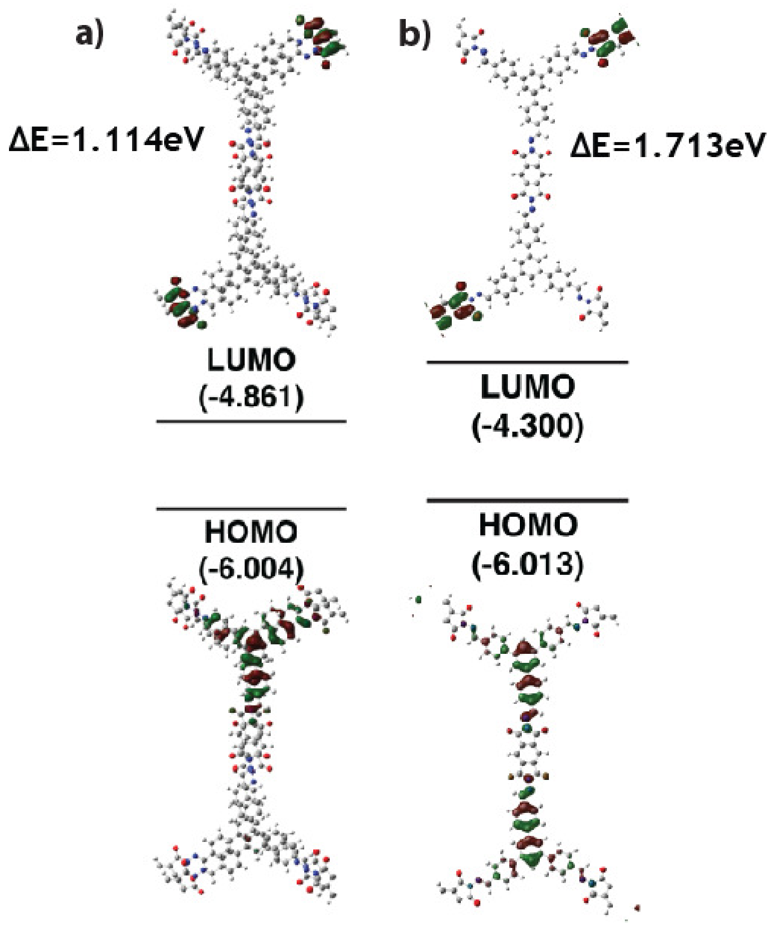

Figure S29: HOMO-LUMO energy profile comparision of a) **TfpBDH-**CONs (two layers) and b) **TfpBDH-**CONs (single layer)**.**

Section S-14: Tyndall Effect

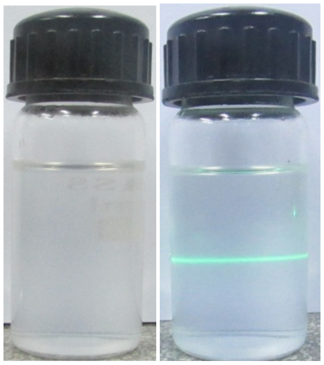

Figure S30: Tyndall effects showed by **TfpBDH-**CONs dispersed in IPA.

Section S-15: Dynamic Light Scattering (DLS)

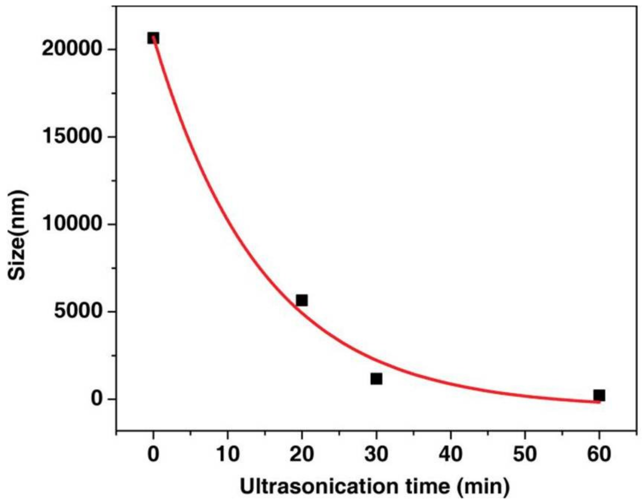

Figure S31: Mean size of the CONs in function of ultra-sonication time, as obtained from DLS.

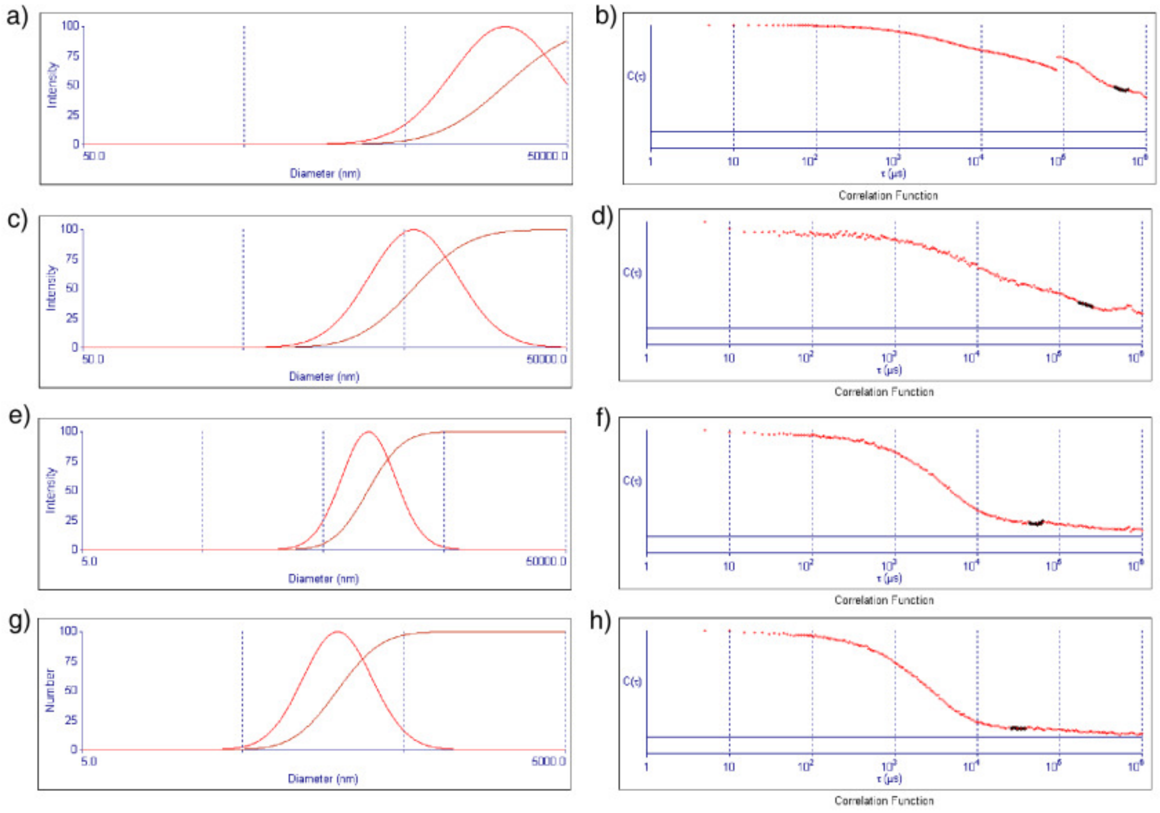

Figure S32: a), c), e) and g) Plots of hydrodynamic diameter with respect to the scattering intensity and b), d), f) and h) Correlation function plots obtained by DLS analysis at differnet sonication time interval.

Section S-16: References

1. J. H. Chong, M. Sauer, B. O. Patrick, M. MacLachlan, J.*Org. Lett.* 2003, **5**, 3823.
2. H. Ghassemi, A. S. Hay, *Macromolecules* 1994, **27**, 3116.
3. P. Rajakumar, M. G. Swaroop, S. Jayavelu, K. Murugesan, *Tetrahedron* 2006, **62**, 12041.
4. M. A. Addicoat, D. E. Coupry, T. Heine, *J. Phys. Chem. A*, 2014, **118**, 9607.
5. Accelrys, Material Studio Release Notes, Release 4.2, Accelrys Software, San Diego 2006.
